# Metabolomic and transcriptomic analysis of *Lycium chinese* and *L. ruthenicum* under salinity stress

**DOI:** 10.1186/s12870-021-03375-x

**Published:** 2022-01-03

**Authors:** Xiaoya Qin, Yue Yin, Jianhua Zhao, Wei An, Yunfang Fan, Xiaojie Liang, Youlong Cao

**Affiliations:** Wolfberry Science Institute, Ningxia Academy of Agriculture and Forestry Sciences / National Wolfberry Engineering Research Center, Yinchuan, 750002 China

**Keywords:** Wolfberry, *Lycium. Chinese*, *Lycium. Ruthenicum*, Salinity stress, Abscisic acid, Flavone, Flavonoid

## Abstract

**Background:**

High soil salinity often adversely affects plant physiology and agricultural productivity of almost all crops worldwide, such as the crude drug known as wolfberry. However, the mechanism of this action in wolfberry is not fully understood yet.

**Results:**

Here in this study, we studied different mechanisms potentially in Chinese wolfberry (*Lycium chinese*, LC*)* and black wolfberry (*L. ruthenicum*, LR*)* under salinity stress, by analyzing their transcriptome, metabolome, and hormone changes. The hormone detection analysis revealed that the ABA content was significantly lower in LR than LC under normal condition, and increased sharply under salinity stress in LR but not in LC. The transcriptome analysis showed that the salinity-responsive genes in wolfberry were mainly enriched in MAPK signaling, amino sugar and nucleotide sugar metabolism, carbon metabolism, and plant hormone signal transduction pathways in LC, while mainly related to carbon metabolism and protein processing in endoplasmic reticulum in LR. Metabolome results indicated that LR harbored higher flavone and flavonoid contents than LC under normal condition. However, the flavone and flavonoid contents were hardly changed in LR, but increased substantially in LC when exposed to salinity stress.

**Conclusions:**

Our results adds ABA and flavone to mechanism understanding of salinity tolerance in wolfberry. In addition, flavone plays a positive role in resistance to salinity stress in wolfberry.

**Supplementary Information:**

The online version contains supplementary material available at 10.1186/s12870-021-03375-x.

## Background

Currently, more than one-third of the world’s agricultural acreage is affected by salinization. Soil salinity is a worsening global problem that impairs plant growth and crop yield, posing serious problems to modern agriculture [1]. Accordingly, perhaps the most efficient way to prevent such crop production losses induced by salinity is to cultivate salt-tolerant plant varieties. Hence, a better understanding of the mechanisms by which plants respond to salt stress becomes imperative, as this will help to improve tolerance to salinity in crops via biotechnological approaches. To achieve this goal, it is imperative to study the salt-tolerance mechanisms of plants native to high-salinity environments, such as wolfberry. For example, the leaves of *Lycium ruthenicum* are notably thickened to adapt to high salinity conditions. Importantly, salinity stress can suppress plants growth and impair their development at multiple scales, such as physiological, phytohormone, and metabolism.

In terms of physiological responses, salinity stress typically induces osmotic stress and ionic imbalance in plants. Osmotic stress accompanied with salinity stress gives rise to the rapid closure of stomata, which reduces the plant’s ability to absorb CO_2_ [2]. Furthermore, the ionic imbalance induced by the excessive accumulation of Na^+^ and Cl^−^ results in ionic toxicity, which does harm to plant and may even kill it by inhibiting the activity of enzymes under salinity stress conditions [3]. Since Na^+^ is similar to K^+^, any surplus Na^+^ would replace K^+^ in some enzymatic reactions to reduce various enzyme activities, such as those involved in primary metabolism, glycolysis, and Calvin cycle [4, 5]. Superabundant Cl^−^ in the shoot tissue can replace the non-selective anion transporters of NO_3_^−^ and SO_4_^2−^, thereby leading to a shortage of key macro-nutrients like N and S in the affected plants [6].

Furthermore, being a versatile signal, reactive oxygen species (ROS) are rapidly induced by salinity stress, mainly in the apoplast, chloroplast, mitochondria, and peroxisomes [7]. The *AtRbohD* and *AtRbohF* genes responsible for ROS production are both up-regulated under high salinity [8]. Several studies have revealed that *AtRbohD* and *AtRbohF* play positive roles in the salinity stress tolerance. ROS production from *AtRbohD* and *AtRbohF* at the early stage of salt stress contributes to lignin formation under saline environment and this reduces oxidative damage to cells [8]. At low concentrations, ROS often act as normal signals in regulating many biological processes, but when in excess they play a harmful role in plant growth, which manifests as lipid peroxidation in cellular membrane, protein denaturation, and impairment of enzymatic activities [9]. The greater ion fluxes across the thylakoid membrane via ion channels, activated by H_2_O_2_, cause thylakoids swelling, leaving the photosynthetic performance of chloroplasts diminished [10, 11].

Many sensors operate along the salt stress-signaling pathway in plant to avoid damage caused by high salinity. High salinity could increase cytosolic Ca^2+^ within just seconds to minutes [12, 13]. Recently, researchers have identified that glycosyl inositol phosphorylceramide (GIPC) sphingolipids directly bind to Na^+^ and regulate the entry of Ca^2+^ into cytosol [14]. Some other proteins also have been reported as mediators in salt-induced Ca^2+^ signaling, namely FERONIA(*FER*), annexin1 (*ANN1*) and plastid K^+^ exchange antiporters (*KEA*s) [15–17]. The cell wall-localized leucine-rich repeat extensions *LRA3*, *LRX4*, and *LRX5* participate in the sensing and relaying of salt stress signals by monitoring the status of cell wall integrity, and function together with secretory peptides *RALF*s and the receptor-like kinase *FER* [18].

High salinity also induces osmotic stress in plants as well as organellar stress such as chloroplast stress. For example, the SNF1-related protein kinase 2 s (*SnRK2*) can be activated by osmotic stress in an ABA-dependent or ABA-independent manner, which contributes to greater inhibition of plant growth and promotes leaf chlorosis under osmotic stress [19–21]. Moreover, the biosynthesis of amino acids, fatty acids, and lipids occurs in chloroplasts [22]. The photosynthetic impairments arising from by damaged chloroplast is a major reason why plant growth is inhibited under salt stress [23, 24]. Most ABA biosynthesis-associated proteins, such as *ABA1*, *ABA4*, and *NCED3*, are localized in chloroplast where most of the steps in ABA biosynthesis also take place, which are required in ABA accumulation that induced by salt stress [25]. As the predominant phytohormone involved in the plant response to salinity stress, ABA increases rapidly and massively in root and leaf tissues within just several minutes [26, 27]. Furthermore, it has been reported that precursors transported from leaves are required for ABA synthesis in roots [28]; the stress-induced augmentation of ABA levels in roots is several fold higher than in leaves [27] and salinity stress is known to induce a significant accumulation of ROS in plant roots [29]. In this respect, ABA can interact with H_2_O_2_ in plant systemic responses to abiotic stresses [30]. For osmotic stress to induce greater H_2_O_2_ production requires *NADPH* oxidase, with the latter was stimulated by ABA [31].

The accumulation of compatible osmolytes helps plants maintain a low intracellular osmotic potential under conditions of high salinity [32, 33], including proline, glycine betaine, sugars, and polyamines, among others [34, 35]. Proline in particular is pivotal for an osmotic adjustment under salt stress; it accumulates through the activation of its biosynthesis pathway and the suppression of its catabolic pathway [36]. Furthermore, proline also acts as a ROS scavenger to attenuate oxidative stress and this assists in stabilizing proteins and membrane structures under high salinity [37–39]. Besides proline, some sugars, namely glucose, fructose, and myoinositol, can also function as signals in plants response to high salinity [40].

Soil salinity will continue to threaten crop production and food security in the future. Therefore, additional research on the dynamics of transcriptome and metabolism networks of plants as they respond to salinity stress is necessarily. Nevertheless, since the ability of plants to tolerate high salinity varies widely among species, this provides an opportunity to identify genes and metabolites that are pivotal for conferring salinity tolerance to plants.

Wolfberry is a genus of perennial shrub (*Lycium* L.), in the Solanceae family, whose distribution in China ranges across Xinjiang, Ningxia, Qinghai, Gansu, and Inner Mongolia. The black wolfberry (*L. ruthenicum*, LR) generally occurs in saline soil or in desert ecosystem, being a typical wild plant that is both drought-resistant and salt-tolerant, which also has high economic and nutritive value in China. Black wolfberry is recognized for its many advantages in cultivation, mainly its resistance to drought and cold, and its tolerance to salinity. Salt stress had negative effects on photosynthesis, Chlorophyll fluorescence, and physiology of Goji berry [41]. Another study consider the mechanism which ABA affects drought resistance in tetraploids and diploids, to understand the physiological and molecular mechanisms that enhance abiotic stress tolerance in polyploid plants [42]. A study in tobacco demonstrated that *LchERF*, a novel ethylene responsive transcription factor from *Lycium chinense*, might confer salt tolerance in transgenic tobacco and mediate various physiological pathways that enhance salt stress tolerance in plants [43]. At the same time, overexpression of the flavanone 3-hydroxylase gene *LcF3H* from *Lycium chinense* enhances drought stress in tobacco, and there is a positive link with endogenous *LcF3H* expression level [44].

However, only few studies have investigated the molecular mechanism underpinning salinity resistance in different wolfberry species. As black wolfberry (*Lycium ruthenicum* Murr., LR) often live in the saline desert, with blade fleshy leaves, which is a characteristic of the salt-tolerant plants. We want to know the advantages of black wolfberry compared to other wolfberry species in living in the salinity soil. While Chinese wolfberry (*Lycium chinese* Mill*.,* LC) is a kind of *Lycium barbarum* which is widely distributed worldwild, also including saline soil. Therefore, in this study, we choose black wolfberry and Chinese wolfberry as research materials, to explore the difference between these two wolfberry species when exposed to salinity stress at transcriptome or metabolom level. The differentially expressed genes and metabolites in LC and LR were analyzed and filtered via transcriptome and metabolism sequencing techniques. Furthermore, complementing this, we also investigated the different responses to salinity stress between Chinese wolfberry and black wolfberry, and,analyzed by examining their transcriptome and metabolom. With these results, the molecular mechanisms of tolerance to salinity stress in black and Chinese wolfberry could be revealed, as well as the key players involved was identified, which will improve our understanding of how these species respond to salinity stress.

## Results

### Phenotypic differences between *L. chinese* and *L. ruthenicum* in response to salinity stress

**Fig. 1:**
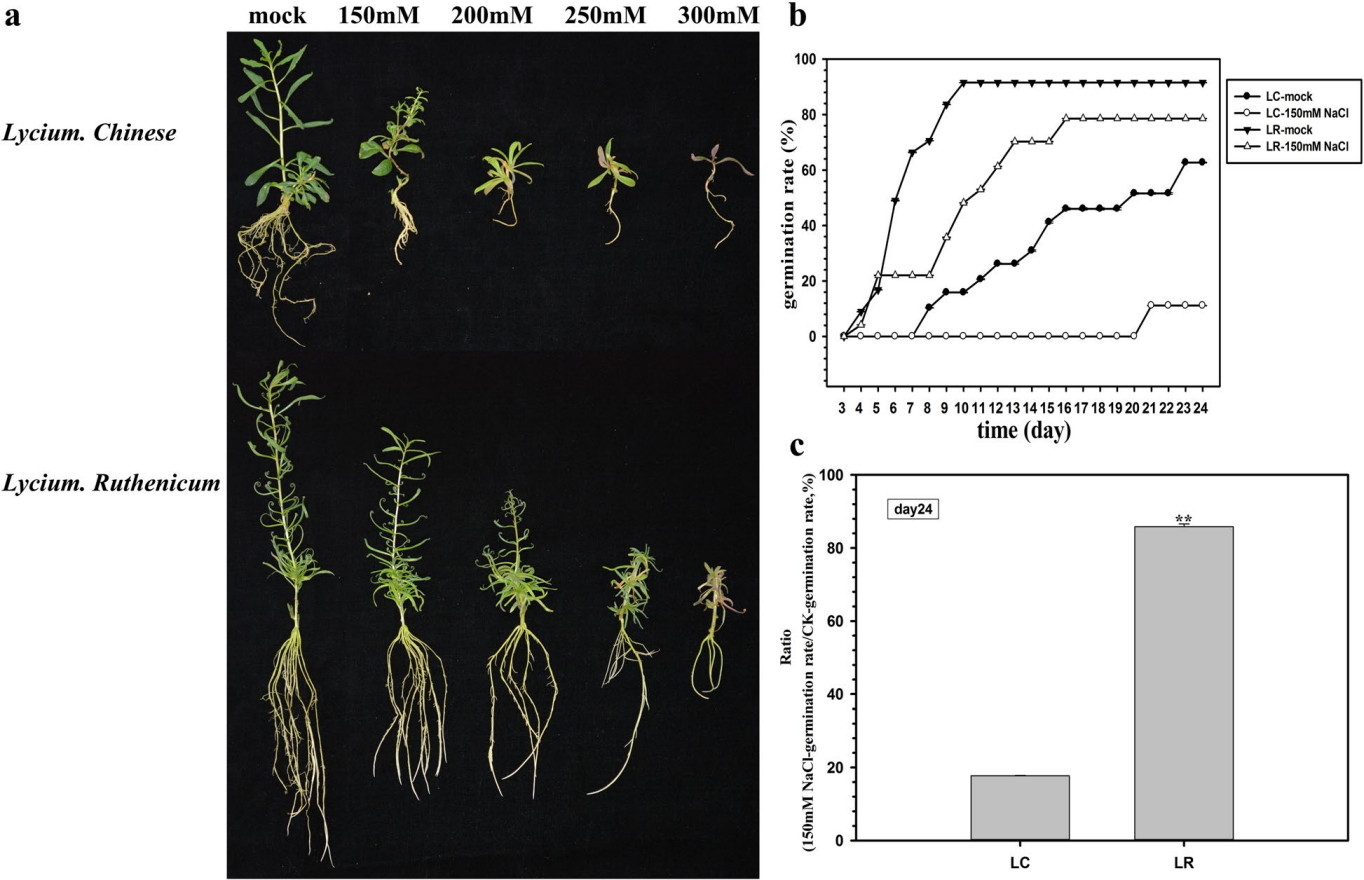
Phenotype analysis of *Lycium chinese* and *L. ruthenicum* under salinity stress. **a** The twig cuttings of *L. chinese* and *L. ruthenicum* were planted in the MS medium containing 0 mM (CK), 150 mM, 200 mM, 250 mM or 300 mM of NaCl. Pictures were taken after three weeks of cultivation. **b** Seed germination rate of *L. chinese* and *L. ruthenicum* under control and 150 mM NaCl concentrations was calculated from day 3 to day 24. **c** The ratio of germination rate 150 mM NaCl/ CK was calculated at day 24 after seeds were sown. * *P* < 0.05, ** *P* < 0.01. *N* = 3. Bars = means ± SEM

To investigate the effect of salinity stress on growth and development of wolfberry, and the possible mechanisms regulating responses to high salinity, Chinese wolfberry (*Lycium. chinese*) and black wolfberry (*Lycium. ruthenicum*) were selected as experimental materials. Firstly, the growth statement of LC and LR was examined, by planting twig cuttings in the Murashige and Skoog (MS) medium containing 0 mM (control), 150 mM, 200 mM, 250 mM, and 300 mM of NaCl. Figure 1a shows the images taken after three weeks cultivation. The growth of wolfberry seedlings was increasingly inhibited by salinity stress, they had fewer and shorter roots under higher salt concentration, and their leaves number was reduced and became more yellowish. For LR, its leaves displayed a sharp yellowish phenomenon and roots were notably shortened under 300 mM NaCl condition; this phenomenon was occoured in LC as well, but became occour from 200 mM NaCl condition and more seriously under 300 mM NaCl. Hence, the capacity to withstand salinity stress was significantly weaker in LC than LR seedlings. Furthermore, the root system of wolfberry usually consists of taproot, lateral root and fibrous root. The taproot is developed from germinated seed, so that only seed-propagating wolfberry plants have taproots. In addition, the seedlings propagated by vegetative propagation, such as cuttage in Fig. 1a, only have lateral roots and fibrous roots, but have no taproots. So that the root growth analyses didn’t carry out continuely.

In addition, the seed germination rate of LC and LR under salinity stress was also tested. There was stress effect but not lethal to seedlings under 150 mM NaCl concentration, and the response differences between different species could be observed, which can be seen from Fig. 1a. Therefore, 150 mM was selected as the concentration in germination experiment. As Fig. 1b and c shown, germination rate was more severely impaired in LC than LR; the germination in LR was higher and occurred sooner than LC under the control condition and 150 mM NaCl concentration. Furthermore, relative to the control group, more sown seeds of LR (86%) germinated than those of LC (18%), indicating the ability to germinate of LC is hindered by salinity stress severely, but not LR.

In addition, the salt content has been determined. As shown in the Fig. S1, the Na^+^ content、K^+^ content and K^+^/Na^+^ ratio has been determined respectivily. The LR leaves have a higher Na^+^ content than LC when under 150 mM NaCl condition, and a smaller K^+^/Na^+^ ratio decrease scale from mock to 150 mM NaCl condition than LC (Figure S1abc). Furthermore, the LC contains a wide decrease of Fv/Fm、a sharp increase of MDA content and GSH content than LR from mock to 150 mM NaCl condition. The LR leaves contain a higher GSH content under mock condition than LC so that possess a stronger antioxidant effect. And the LR accumulate more proline under salinity condition than LC to defence peroxidating (Figure S1defgh).

### Hormone changes in wolfberry in response to salinity stress

**Fig. 2:**
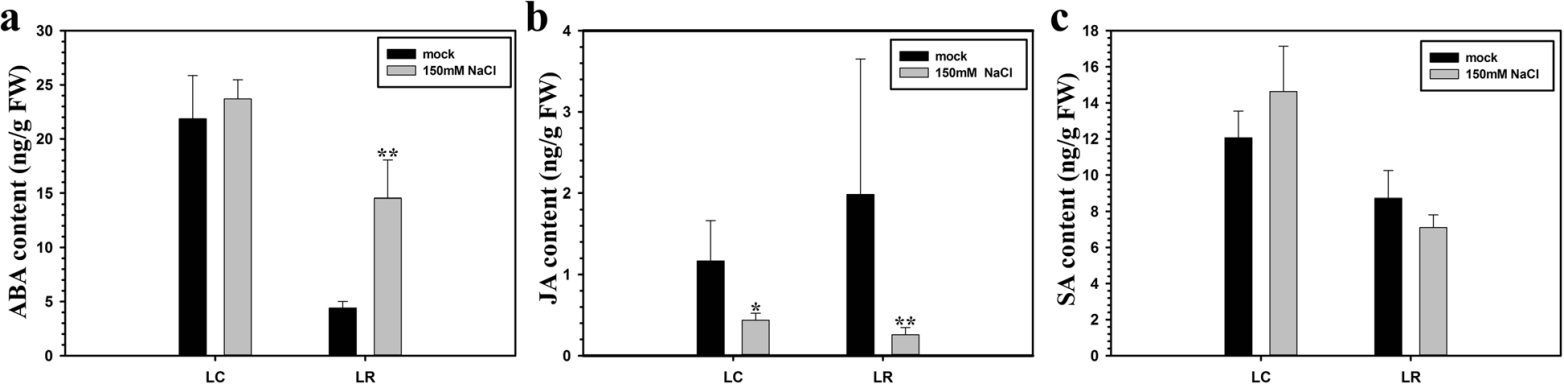
Hormonal variation of *Lycium chinese* and *L. ruthenicum* in response to salinity stress. Quantification of ABA (Abscisic acid), JA (Jasmonic acid), and SA (Salicylic acid) contents of *L.* chines*e* and *L. ruthenicum* leaves at indicated time points after NaCl treatment. ABA content in **a**, JA content in **b**, and SA content in **c**. Data represent the means ± SEM of three replicates. Asterisks indicate a significant difference from mock (non-stressed treatment at the same time point (N = 3, Student’s *t*-test: * P < 0.05, ** P < 0.01)

To further explore the differences between LC and LR in resistance to salinity stress, the abscisic acid (ABA), jasmonic acid (JA), and salicylic acid (SA) content in leaves of LC and LR under control and 150 mM NaCl conditions were tested. As Fig. 2 shows, for the control group, the ABA content in LR was significantly lower than that in LC. Compared with the control condition, 150 mM NaCl treatment induced the ABA content slightly increase in LC leaves, whereas a significantly large amount of ABA accumulated in the leaves of LR (Fig. 2a). Further, the JA content was significantly higher in leaves of LR than LC under control condition, though JA accumulation was significantly reduced by salinity stress, more in LR than LC (Fig. 2b). The SA content in the leaves of LR was lower than that in LC under control condition, but was not significantly affected by the salinity treatment in both species (Fig. 2c). These results indicated that the resistance to high salinity stress in LR was driven by accumulating ABA while reducing JA content.

### The transcriptomic of LC and LR responsing to salt stress

**Fig. 3:**
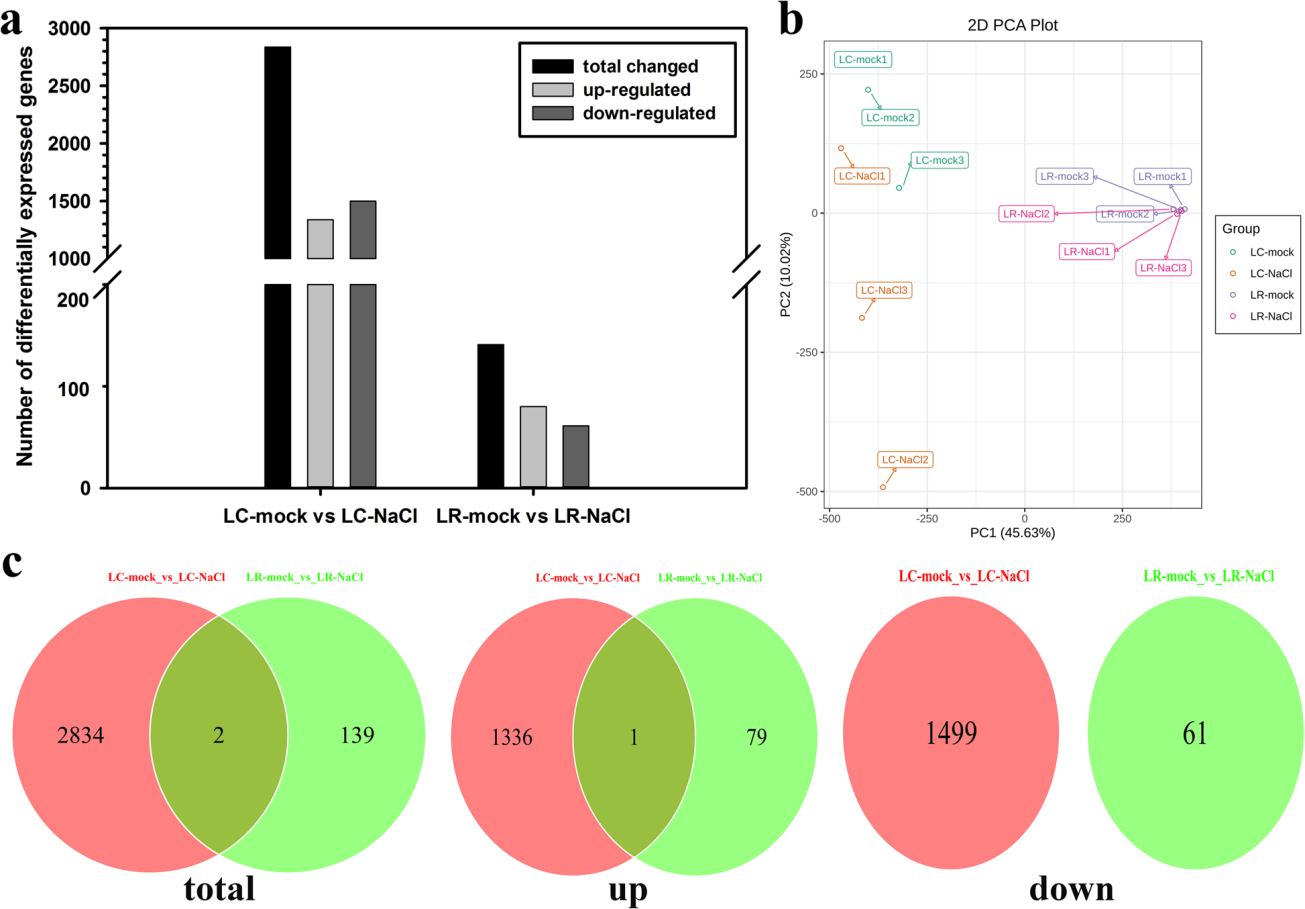
Transcriptome data of *Lycium chinese* and *L. ruthenicum* leaves in response to salinity stress. **a** Number of differentially expressed genes (DEGs) in *L. chinese* and *L. ruthenicum* under salinity stress. **b** PCA (Principal component analysis) clustering based on the plants’ transcriptome data. **c** Venn diagrams of DEGs between normal and salinity stress conditions in *L. chinese* and *L. ruthenicum*

To better understand the molecular basis of salinity stress responses in LC and LR, we carried out transcriptomic sequencing and analyzed different expressed genes (DEGs) in LC and LR under control and salinity conditions. A total of 2836 DEGs were detected in LC under salinity stress compared with the control group, in which 1337 genes were up-regulated and 1499 genes down-regulated. For LR, however, only 141 genes were differentially expressed when treated with high salinity, in which 80 genes were up-regulated and 61 genes were down-regulated (Fig. 3a). To identify the key determinate factors of the transcriptome, PCA was performed on the genes of the two species under control and salinity treatment conditions. The first two principal components (PC1, PC2) were able completely distinguish our combinations of species and treatment (i.e., 2 species × 2 treatment levels [mock and 150 mM salinity concentration]). The PCA shows a clear separation between different species along PC1 and the separation of treatment can be observed along PC2. In addition, the three biological replicates were projected closely in the ordination space, which suggested a good correlation between replicates (Fig. 3b). A Venn diagram was used to analyze and display the differences between variation genes of LC and LR under salinity stress respectively. As depicted in Fig. 3c, group LC-mock vs. LC-NaCl and group LR-mock vs. LR-NaCl only shared two changed genes in total under salinity stress, and only 1 common regulated gene in the up venn diagram and no common regulated gene in the down venn diagram. It is because that the common regulated genes in the total venn diagram refers to the genes whose expression level has changed in the both groups, no matter up-regulated or down-regulated. But the common genes in the up venn diagram only refers to the up-regulated genes and so does in down venn diagram. In addition, the ferritin-3 gene (*Cluster-40,571.167017*) was up-regulated both in group LC-mock vs. LC-NaCl and group LR-mock vs. LR-NaCl, and the heat shock cognate gene (*Cluster-40,571.121975*) was up-regulated in group LC-mock vs. LC-NaCl but down-regulated in group LR-mock vs. LR-NaCl.

### KEGG enrichment of DEGs in LC and LR under salinity stress

**Fig. 4:**
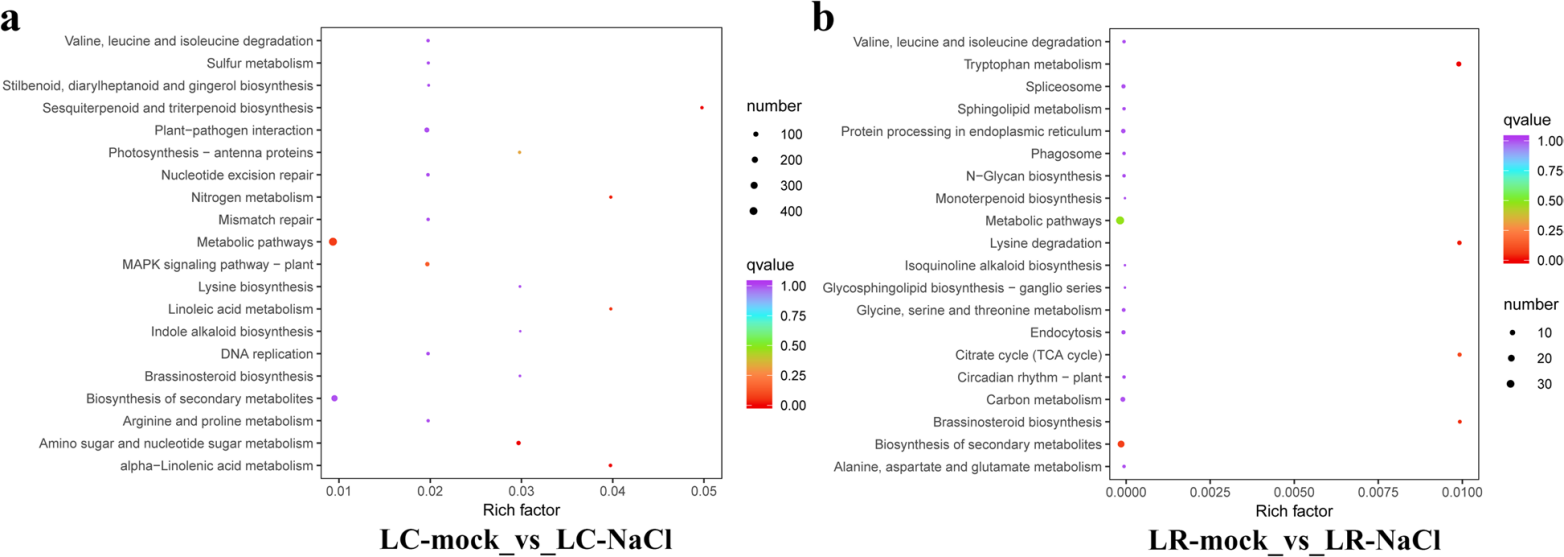
Statistics of KEGG enrichment analysis of DEGs (different expressed genes) in *Lycium chinese* (LC) and *L. ruthenicum* (LR) under salinity stress. **a** Group LC-mock vs. LC-NaCl. **b** Group LR-mock vs. LR-NaCl

Evidently, as shown in Fig. 4a, for LC-mock vs. LC-NaCl, the differential genes between the control and salinity condition in LC are mainly enriched in metabolic pathways (48.57%), biosynthesis of secondary metabolites (22.81%), plant–pathogen interaction pathway (10.26%), MAPK signaling pathway (6.16%), amino sugar and nucleotide sugar metabolism pathway (6.16%), carbon metabolism (5.47%), and plant hormone signal transduction (5.02%). In the group LR-mock vs. LR-NaCl (Fig. 4b), the DEGs between control and salinity condition in LR are mainly enriched in metabolic pathways (60%), biosynthesis of secondary metabolites (38.18%), carbon metabolism, protein processing in endoplasmic reticulum, spliceosome, tryptophan metabolism, and lysine degradation.

### Dynamic transcriptome analysis in LC and LR in response to salinity stress

**Fig. 5:**
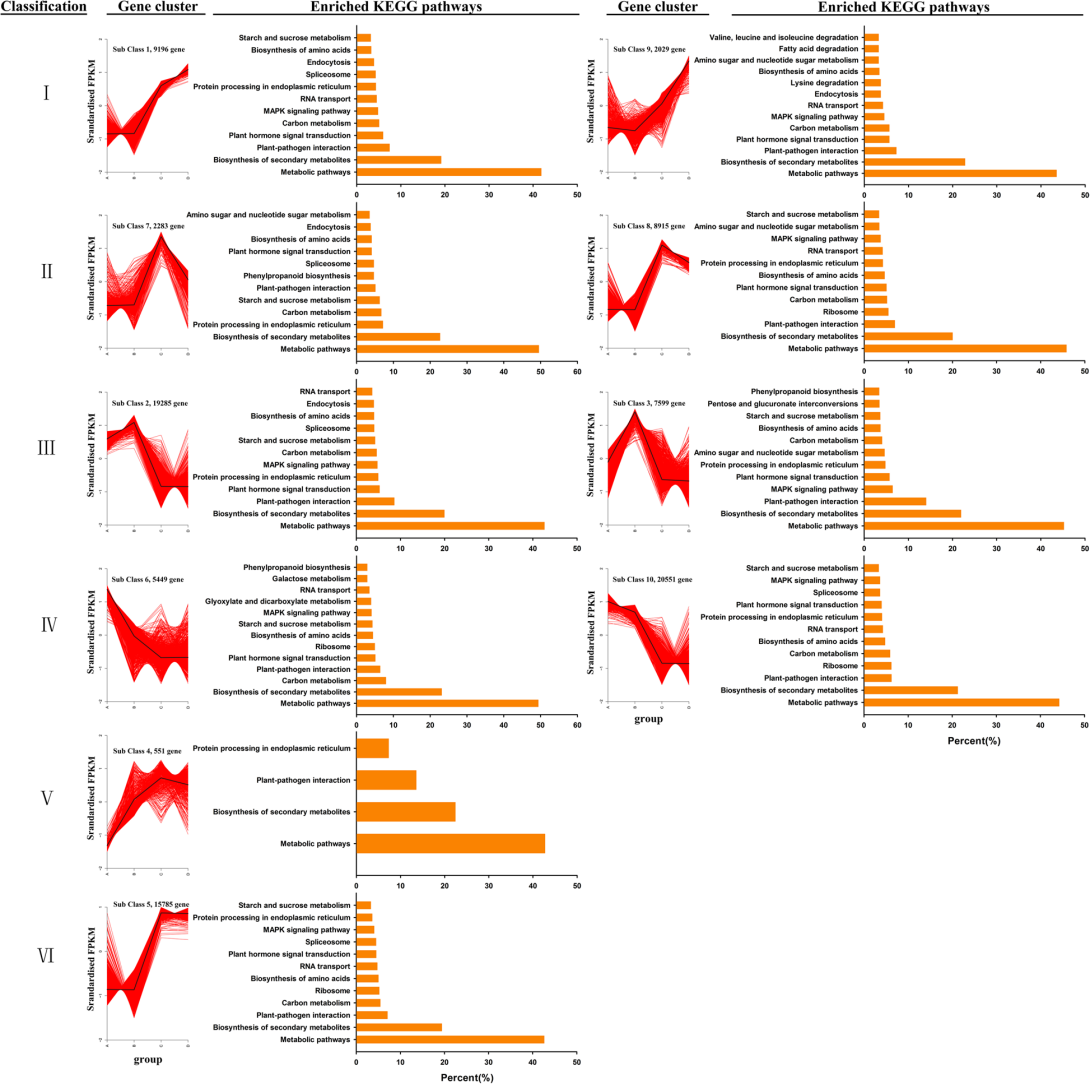
K-means transcriptomic analysis of significant DEGs in *Lycium chinese* and *L. ruthenicum* under control and salinity stress conditions. The DEGs are divided into 10 groups, which are classified into six types. The top 10 KEGG pathways in each group are listed on the corresponding right panel

To study the genes expression patterns in LC and LR under mock and salinity conditions, a K-means cluster analysis was performed, in which the expression patterns of genes in LC-mock, LC-NaCl, LR-mock, and LR-NaCl groups were classified into 10 subclasses, which were then roughly divided into six categories (Fig. 5). The first category was class of genes that showed no regulation change when subjected to salinity stress compared with the background condition in LC, yet they showed a trend of up-regulation in LR (subclass1, subclass9). By contrast, the second category was a class of genes whose regulation levels also unchanged under salinity stress (compared with background condition) in LC but whose tendency was down-regulated in LR under salinity stress (subclass7, subclass8). The third category of genes featured an up-regulated expression trend under salinity stress in LC, which remained unchanged in LR under salinity stress (subclass2, subclass3). The genes in the fourth category were down-regulated in LC while mostly unchanged in LR under salinity stress (subclass6, subclass10). Concerning the fifth category genes, they were up-regulated in LC yet down-regulated in LR when exposed to high salinity stress (subclass4). The expression level of the sixth category genes had not induced by salinity stress in either LC or LR (subclass5).

KEGG pathway enrichment analysis was carried out for the 10 subclasses. These results demonstrated that these DEGs were mainly involved in metabolic pathways and biosynthesis of secondary metabolites pathways. In the first category, the first five pathways in both subclass1 and subclass9 are plant–pathogen interaction, plant hormone signal transduction, carbon metabolism, MAPK signaling pathway, and RNA transport. In the second category, the first five pathways in subclass7 are protein processing in endoplasmic reticulum, carbon metabolism, starch and sucrose metabolism, plant–pathogen interaction, and phenylpropanoid biosynthesis; the first five pathways in subclass8 are plant–pathogen interaction, ribosome, carbon metabolism, plant hormone signal transduction, and biosynthesis of amino acids. In the third category, the first five pathways in subclass2 and subclass3 are plant–pathogen interaction, plant hormone signal transduction, protein processing in endoplasmic reticulum, MAPK signaling pathway, and carbon metabolism. In the fourth category, the first five pathways in subclass6 and subclass10 are carbon metabolism, plant hormone signal transduction, biosynthesis of amino acids, starch and sucrose metabolism, and MAPK signaling pathway. In the fifth category, the main enriched pathways in subclass4 are plant–pathogen interaction, and protein processing in endoplasmic reticulum. In the sixth category, the main enriched pathways in subclass5 are plant–pathogen interaction, carbon metabolism, ribosome, biosynthesis of amino acids, and RNA transport.

### Metabolomic analysis of LC and LR in response to salinity stress

**Fig. 6:**
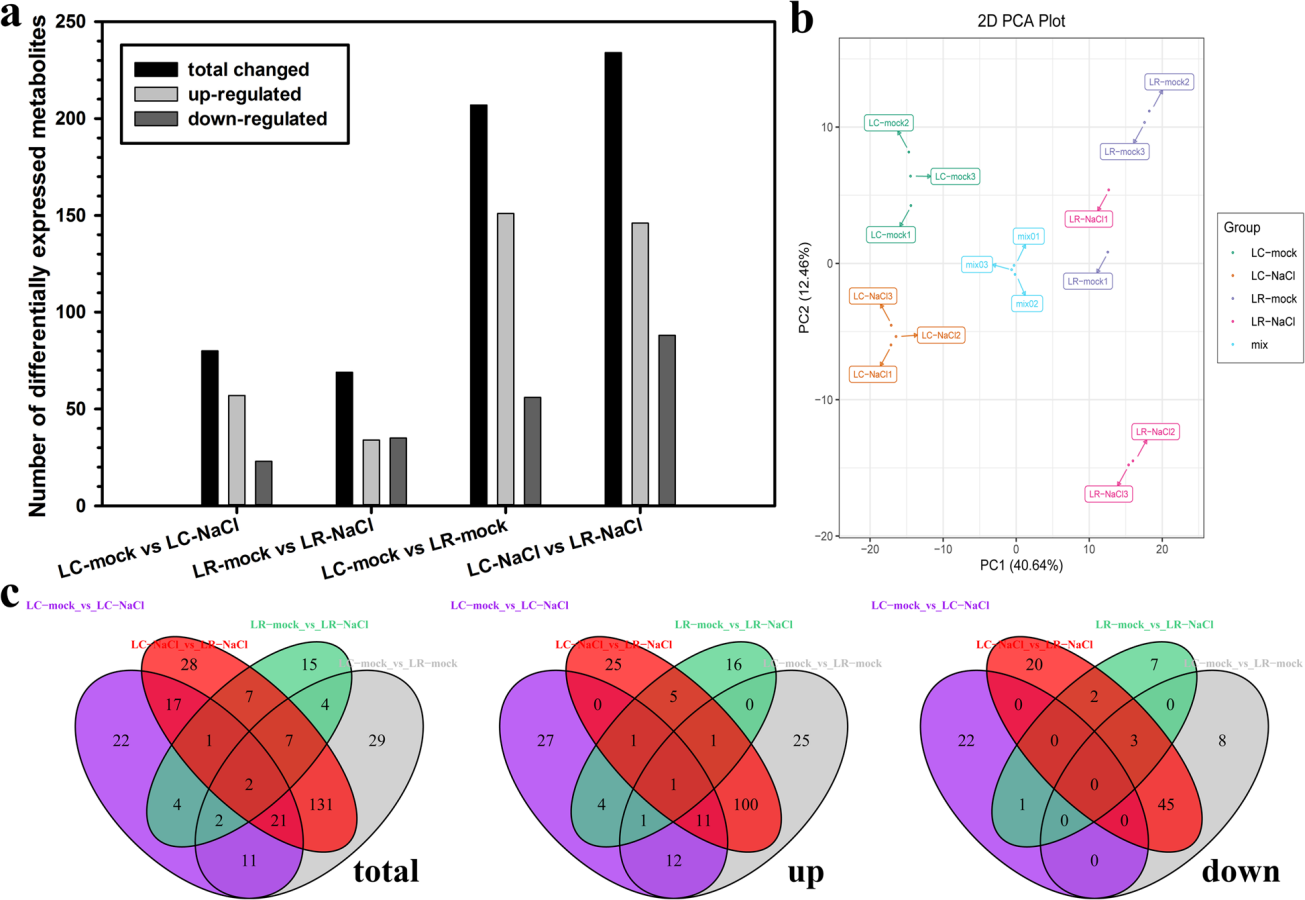
Metabolome analysis of *Lycium chinese* and *L. ruthenicum* in response to salinity stress. **a** Number of differential expressed metabolites in *L. chinese* and *L. ruthenicum* under salinity stress. **b** PCA (Principal component analysis) clustering based on metabolome data. **c** Venn diagrams of the different expressed metabolites between control and salinity stress conditions in *L. chinese* and *L. ruthenicum*

Nextly, the metabolites of Chinese wolfberry and black wolfberry under salinity stress were detected, and the difference of the metabolites between species or conditions were analyzed. As Fig. 6a shows, the expression level of 80 metabolites were changed in LC under salinity stress, in which 57 were up-regulated and 23 were down-regulated. The expression levels of 69 metabolites were changed in LR under salinity stress, in which 34 were up-regulated and 35 were down-regulated. Compared with LC, 207 metabolites were differentially expressed in the leaves of LR under control condition, in which 151 were up-regulated and 56 were down-regulated. In all, 234 metabolites were differentially expressed in the leaves between LC and LR under salinity stress, of which 146 were up-regulated and 88 were down-regulated. The PCA of the metabolites in the control group and the salinity group of LC and LR showed that PC1 and PC2 could completely distinguish the four combinations of species and treatment (Fig. 6b). In Fig. 6c, the difference in metabolites change between different comparative groups was summarized (using a Venn diagram). Groups LC-mock vs. LC-NaCl and LR-mock vs. LR-NaCl shared eight metabolites with common changes, in which seven were up-regulated, and one was down-regulated. Compared with LC-mock vs. LR-mock, the group LC-NaCl vs. LR-NaCl had 161 metabolites featuring the same change tendency, of which 113 were up-regulated and 48 were down-regulated.

### The different metabolites enrichment analysis in LC and LR under salinity stress

**Fig. 7:**
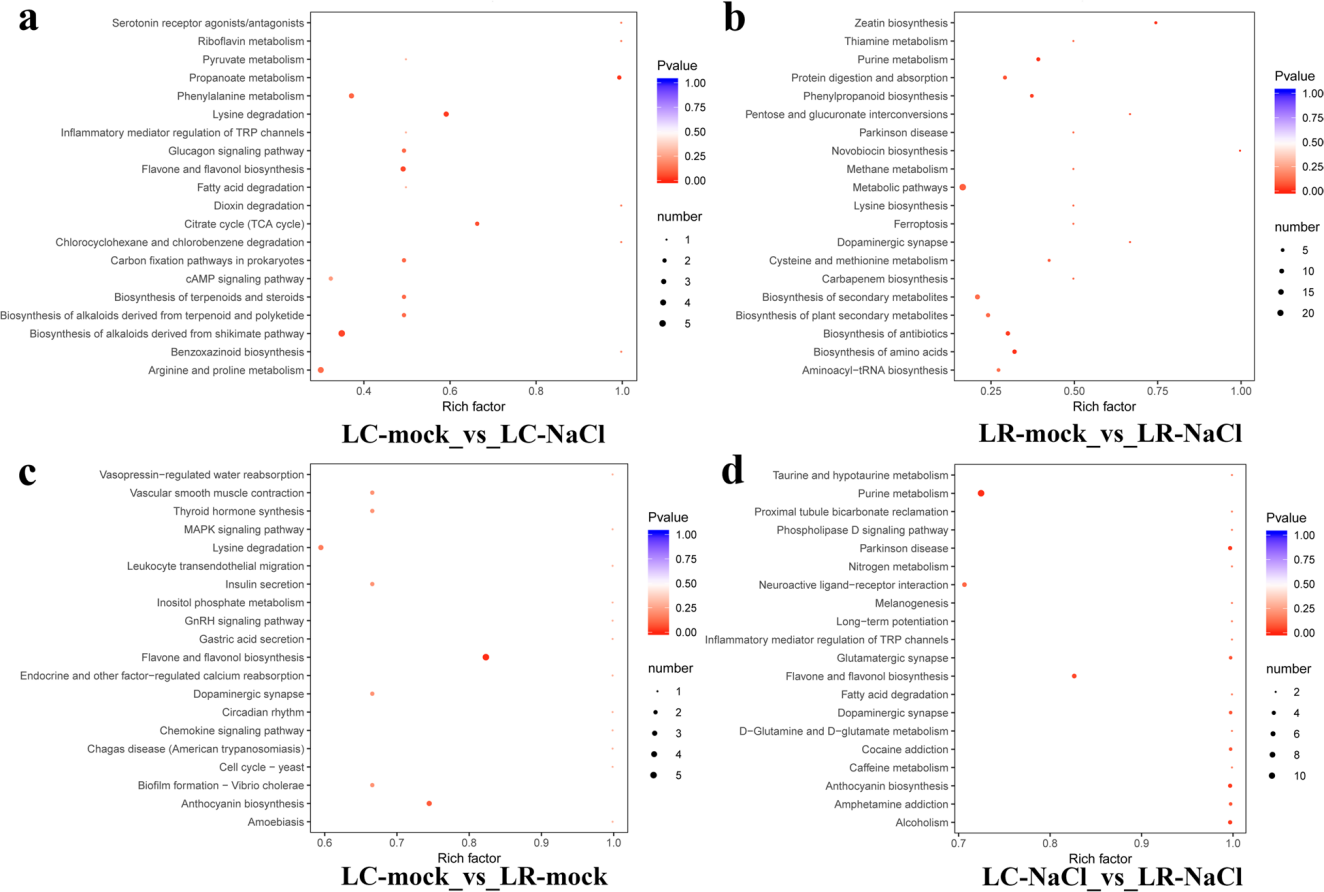
KEGG pathway enrichment of differential expressed metabolites in *Lycium chinese* (LC) and *L. ruthenicum* (LR) under normal and salinity stress conditions. **a** Group LC-mock vs. LC-NaCl. **b** Group LR-mock vs. LR-NaCl. **c** Group LC-mock vs. LR-mock. **d** Group LC-NaCl vs. LR-NaCl

The metabolites in the four comparison groups (LC-mock vs. LC-NaCl, LR-mock vs. LR-NaCl, LC-mock vs. LR-mock, LC-NaCl vs. LR-NaCl) were enriched by KEGG, with the results summarized in Fig. 7. All the metabolites were mainly enriched in metabolic pathways and biosynthesis of secondary metabolites pathways, followed by a detailed analysis of other enrichment pathways. As seen in Fig. 7a, in the group LC-mock vs. LC-NaCl, the changes in metabolites induced by salinity stress in LC mainly concerned these pathways: microbial metabolism in diverse environments, biosynthesis of alkaloids derived from shikimate pathway, biosynthesis of phenylpropanoids, arginine and proline metabolism, and flavone and flavonol biosynthesis. In Fig. 7b, in the group LR-mock vs. LR-NaCl, the metabolites variation induced by salinity stress in the leaves of LR were mainly enriched in the following pathways: biosynthesis of amino acids, purine metabolism, cysteine and methionine metabolism, biosynthesis of amino acids, and protein digestion and absorption. In the group LC-mock vs. LR-mock (Fig. 7c), the different metabolites between leaves of LC and LR under the control condition were mainly concentrated in five pathways: microbial metabolism in diverse environments, pyrimidine metabolism, purine metabolism, flavone and flavonol biosynthesis, and biosynthesis of phenylpropanoids. Finally, in the LC-NaCl vs. LR-NaCl group (Fig. 7d), the disparity in leaf metabolites between LC and LR under salinity pressure mainly arose in the following pathways: microbial metabolism in diverse environments, purine metabolism, tryptophan metabolism, phenylpropanoid biosynthesis, and flavone and flavonol biosynthesis.

### The top 20 differentially expressed metabolites in LC and LR

**Fig. 8:**
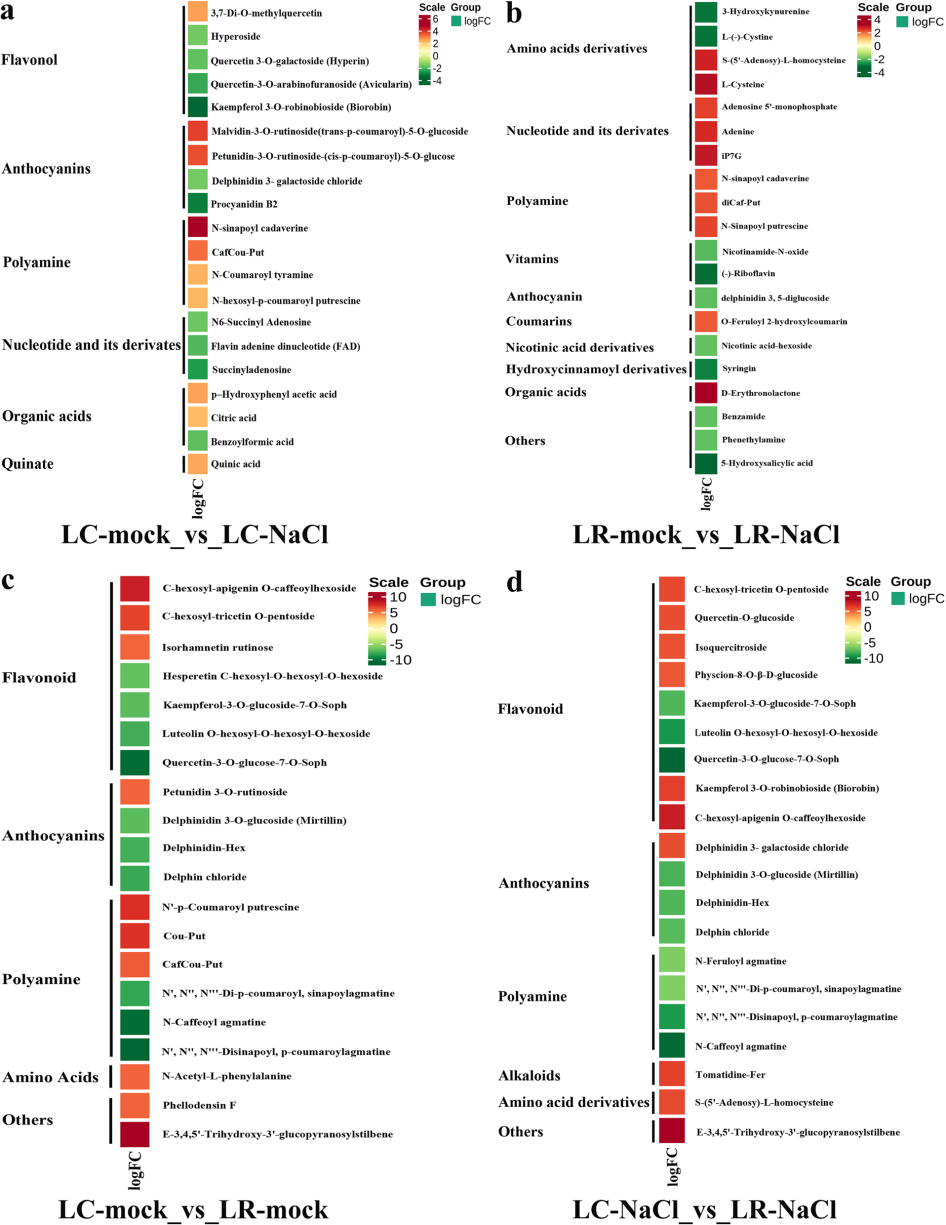
Heatmap of the top 20 significantly differential expressed metabolites in *Lycium chinese* (LC) and *L. ruthenicum* (LR) under control and salinity stress conditions. **a** Group LC-mock vs. LC-NaCl. **b** Group LR-mock vs. LR-NaCl. **c** Group LC-mock vs. LR-mock. **d** Group LC-NaCl vs. LR-NaCl

The top 20 differentially expressed metabolites with more significant log_2_FC whose expression pattern matched their related genes in the four comparison groups (LC-mock vs. LC-NaCl, LR-mock vs. LR-NaCl, LC-mock vs. LR-mock, LC-NaCl vs. LR-NaCl) are shown in Fig. 8. In the group LC-mock vs. LC-NaCl, the highly ranked metabolites were linked to flavonol, anthocyanins, polyamine, nucleotide and its derivatives, organic acids and quinate. More specifically, most flavonols, including hyperoside, hyperin, avicularin, and biorobin, were down-regulated in LC when exposed to salinity. In the anthocyanins classification, malvidin-3-O-rutinoside-5 -O-glucosides were up-regulated, while both delphinidin 3-galaactoside chloride and procyanidin B2 were down-regulated. The polyamines were up-regulated, while the nucleotide and its derivatives were down-regulated (Fig. 8a). In the group LR-mock vs. LR-NaCl, the primarily changed metabolites were associated with amino acids derivatives, nucleotide and its derivatives, polyamine, vitamins, anthocyanin, coumarins, nicotinic acid derivatives, hydroxycinnamoyl derivatives, and organic acids. In greater detail, the amino acids derivatives, 3-hydroxykynurenine and L-(−)-cystine were all down-regulated, whereas S-(5′-adenosy)-L- homocysteine and L-cysteine were both up-regulated during salinity stress. Nucleotide and its derivatives, such as adenosine 5′-monophosphate, adenine, and iP7G, along with the polyamines, such as N-sinapoyl cadaverine, diCaf-put, and N-sinapoyl putrescine, in addition to the organic acids like D-erythronolactone, were all up-regulated in LR when exposed to high salinity. Furthermore, some vitamins, namely nicotinamide-N-oxide and (−)-riboflavin, were down-regulated in LR during salinity stress (Fig. 8b). In the group LC-mock vs. LR-mock, under background condition, the main differential metabolites found were flavonoid, anthocyanins, and polyamine. Some flavonoids were up-regulated in LR compared with LC, like C-hexosyl-apigenin O-caffeoylhexoside, C-hexosyl-tricetin O-pentoside and isorhamnetin rutinose, but others were evidently down-regulated, such as hesperetin C-hexosyl-O-hexosyl-O-hexoside, kaempferol-3-O-glucoside-7-O-soph, luteolin O-hexosyl-O-hexoside and quercetin-3-O-glucose-7-O-soph (Fig. 8c). In the group LC-NaCl vs. LR-NaCl, the major differential metabolites in LR compared with LC under salinity stress were related to flavonoid anthocyanins and polyamine. Most of the gathered anthocyanins and polyamines were down-regulated in LR compared with LC under high salinity. In the flavonoid classification (Fig. 8d), some were up-regulated in LR compared with LC, such as C-hexosyl-tricetin O-pentoside, quercetin-O-glucoside, isoquercitroside, physcion-8-O-β-D-glucoside, biorobin and C-hexosyl-apigenin O-caffeoylhexoside—while several other flavonoids were down-regulated in LR compared with LC under salinity stress (such as kaempferol-3-O-glucoside-7-O-soph, luteolin O-hexosyl-O-hexosyl-O-hexoside, and quercetin-3-O-glucose-7-O-soph).

### KEGG pathway enrichment in DEGs and different expressed metabolites in LC and LR

**Fig. 9:**
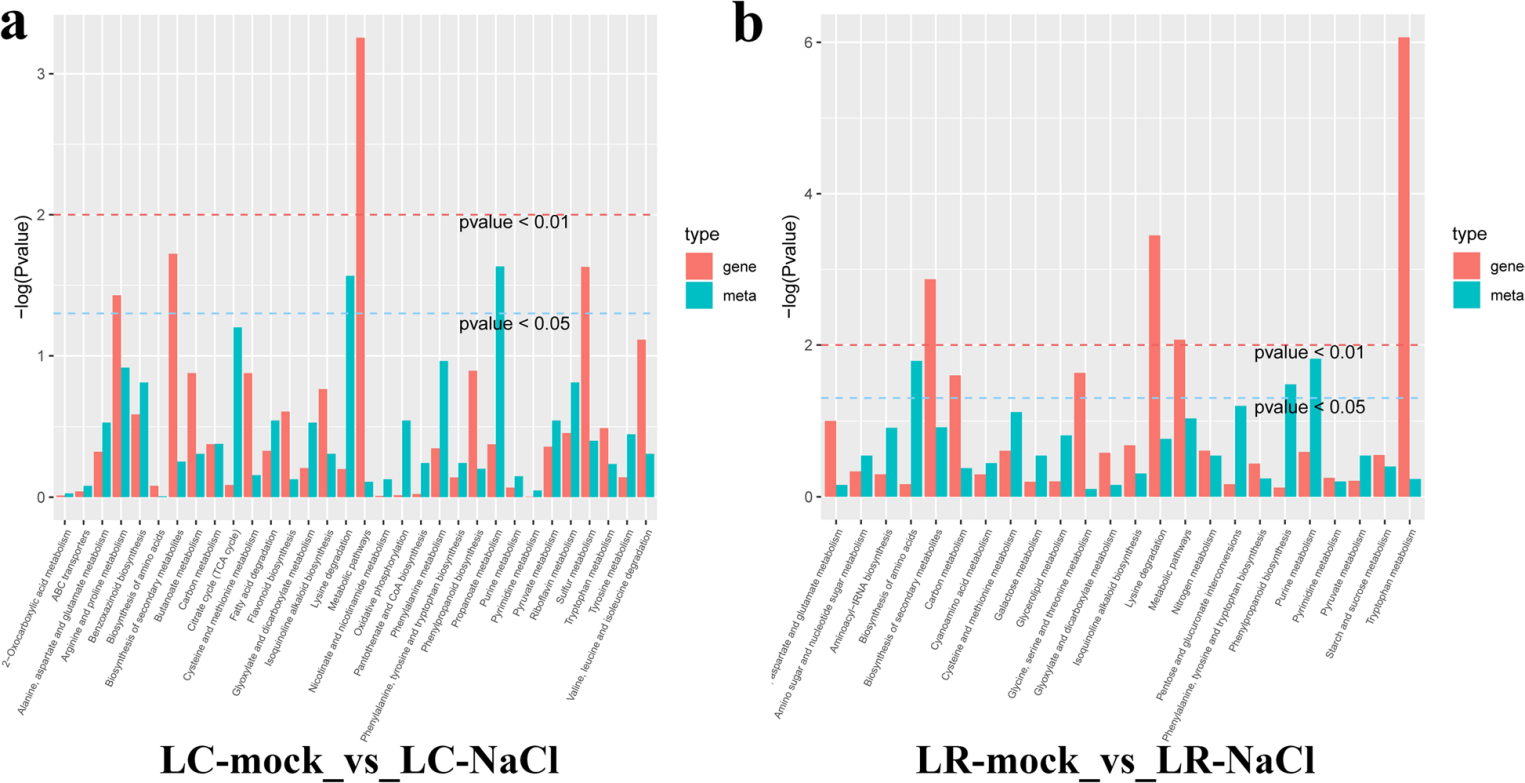
KEGG pathway enrichment (histogram of *P*-values) analysis of *Lycium chinese* (LC) and *L. ruthenicum* (LR) under normal and salinity stress conditions. **a** Group LC-mock vs. LC-NaCl. **b** Group LR-mock vs. LR-NaCl. The abscissa represents metabolic pathways, the ordinate represents enriched P-values, expressed as –log(P-value); the red and green columns respectively represents the differential expressed genes and metabolites

According to the above KEGG enrichment analysis, a histogram was drawn to show the common pathways in which DEGs and differential expressed metabolites were highly enriched. As Fig. 9 shows, in which a taller ordinate column corresponds to greater enrichment. In group LC-mock vs. LC-NaCl (Fig. 9a), the metabolic pathways distinguished by a simultaneously higher enrichment of DEGs and differential metabolites are arginine and protein metabolism, benzoxazinoid biosynthesis, and riboflavin metabolism. In group LR-mock vs. LR-NaCl (Fig. 9b), the corresponding metabolic pathways are lysine degradation pathway, nitrogen metabolism, and purine metabolism.

### Flavonoid metabolism in LC and LR under salinity stress

**Fig. 10:**
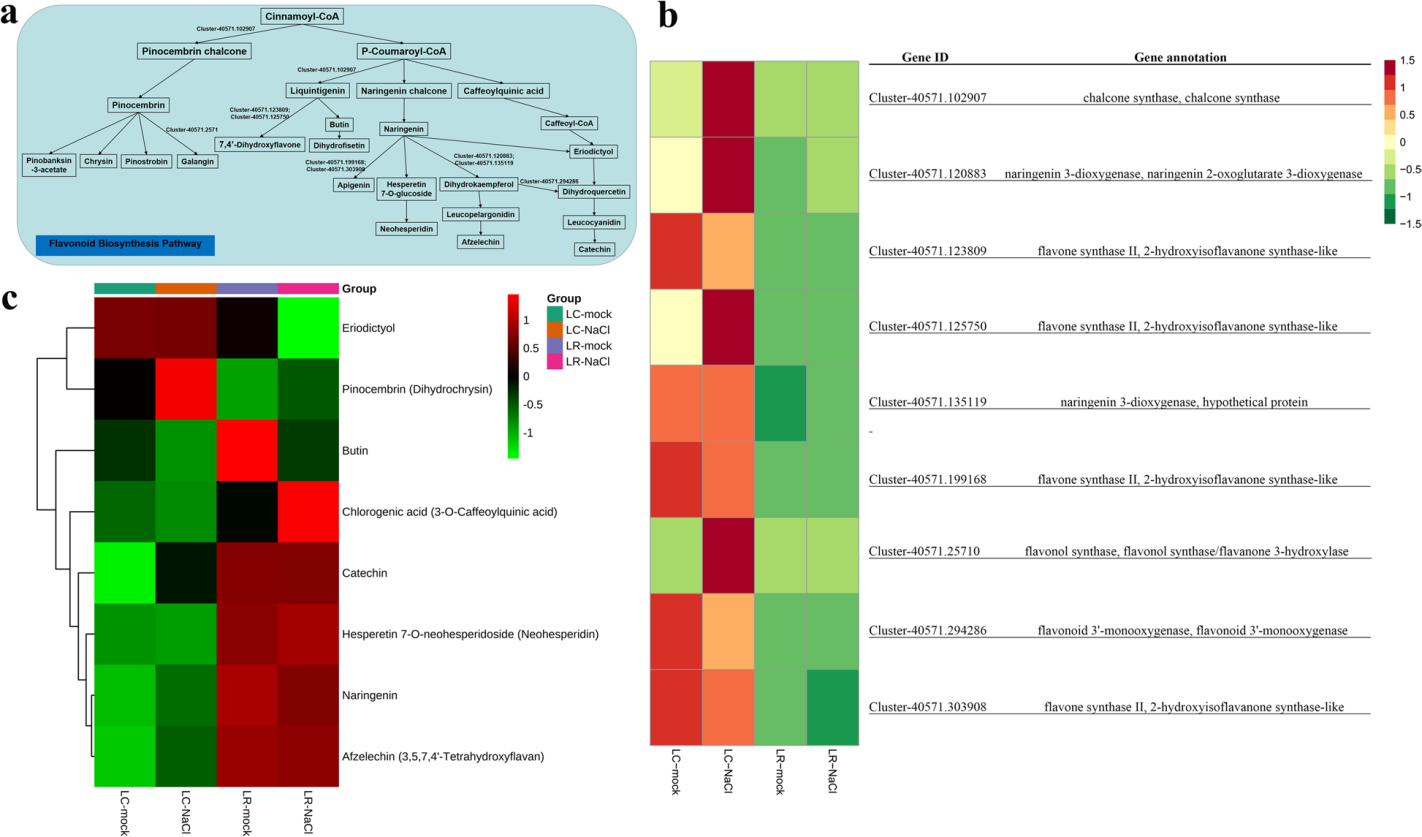
Adaptive changes in flavonoid metabolism in *Lycium chinese* and *L. ruthenicum* under salinity stress. **a** The flavonoid biosynthesis pathway. **b** Heatmap of DEGs (differentially expressed genes) involved in the flavonoid metabolic pathway. **c** Heatmap of differential expressed metabolites in the flavonoid metabolic pathway

Flavonoid metabolism plays an important role in protecting plants against adverse effects of salinity stress. Figure 10a illustrates the flavonoid biosynthesis pathway, for which the marked genes were analyzed in Fig. 10b. The expression profiles of almost all these marked genes in the flavonoid metabolism pathway had a pattern of lower abundance in LR than LC, either under the control condition or salinity stress. Specifically, the genes encoding chalcone synthase (*Cluster-40,571.102907*), flavone synthase II, 2-hydroxyisoflavanone synthase-like (*Cluster-40,571.125750*), and flavonol synthase, and flavonol synthase/flavanone 3-hydroxylase (*Cluster-40,571.25710*) were apparently up-regulated in LC but not in LR when exposed to salinity stress. However, the genes for flavone synthase II, 2-hydroxyisoflavanone synthase-like (*Cluster-40,571.123809*), flavone synthase II, 2-hydroxyisoflavanone synthase-like (*Cluster-40,571.199168*), and flavonoid 3′-monooxygenase, flavonoid 3′-monooxygenase (*Cluster-40,571.294286*) were all not regulated in LR yet down-regulated in LC under salinity stress. Moreover, the genes encoding naringenin 3-dioxygenase and naringenin 2-oxoglutarate 3-dioxygenase (*Cluster-40,571.120883*) were up-regulated in both LC and LR under salinity stress, while naringenin 3-dioxygenase (*Cluster-40,571.135119*) was up-regulated in LR but not regulated in LC. Furthermore, the genes of flavone synthase II, 2-hydroxyisoflavanone synthase-like (*Cluster-40,571.303908*) were down-regulated both in LR and LC under salinity stress (Fig. 10b). Interestingly, most of the changed metabolites in the flavonoid biosynthesis pathway persisted in higher abundance in LR but stay at a lower level in LC under salinity stress, including butin, catechin, neohesperidin, naringenin, and afzelecin, in which, butin was down-regulated both in LR and LC, catechin was non-regulated in LR but up-regulated in LC, neohesperidin was up-regulated in LR but down-regulated in LC, naringenin was down-regulated in LR but up-regulated in LC, and afzelechin was non-regulated in LR but up-regulated in LC. Apart from those metabolites, eriodictyol remained at a lower level in LR than LC, yet it was down-regulated in LR though not regulated in LC. While pinocembrin also occurred at a lower level in LR than LC, it was up-regulated in both LR and LC. In contrast, chlorogenic acid was generally higher in LR than LC, and it was up-regulated in LR but down-regulated in LC (Fig. 10c).

### Alterations in flavone and flavonol metabolism in LC and LR under high salinity

**Fig. 11:**
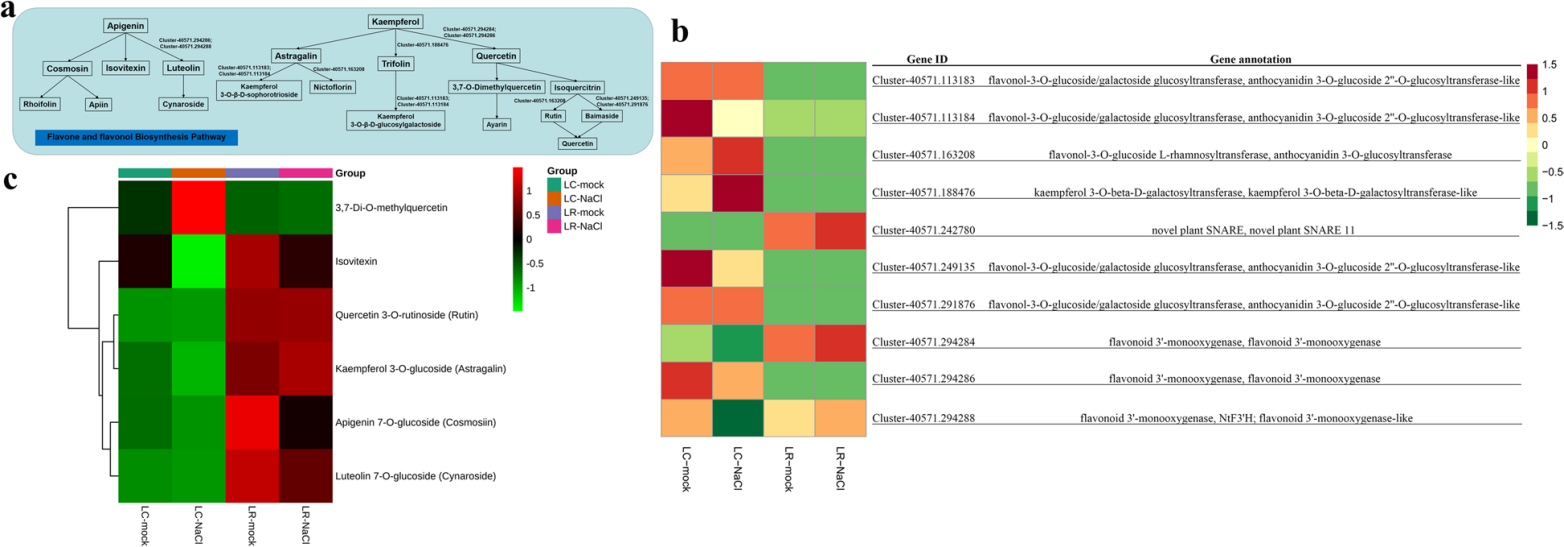
Alterations to flavone and flavonol metabolism in *Lycium chinese* and *L. ruthenicum* under salinity stress. **a** The flavone and flavonol biosynthesis pathway. **b** Heatmap of DEGs (differentially expressed genes) involved in the flavone and flavonol metabolic pathway. **c** Heatmap of differential expressed metabolites in the flavone and flavonol metabolic pathway

It is noteworthy that the flavone and flavonol biosynthesis pathway was enriched significantly in both transcriptomic and metabolomic data of wolfberry plants. The flavone and flavonol biosynthesis pathway appears in Fig. 11a, and the marked expression pattern of relative genes are detailed in Fig. 11b. This revealed that most of the genes involved in flavone and flavonol biosynthesis pathway stay constitutively expressed at low level in LR but at a higher level in LC, such as flavonol-3-O-glucoside glucosyltransferase (*Cluster-40,571.113183*) and galactoside glucosyltransferase (*Cluster-40,571.291876*), although both their expression levels went unchanged during salinity stress in both species. For glucosyltransferase (*Cluster-40,571.113184*), flavonol-3-O-glucoside galactoside glucosyltransferase (*Cluster-40,571.249135*), and flavonoid 3′-monooxygenase (*Cluster-40,571.294286*), their transcription levels were higher in LC and lower in LR, and down-regulated in LC but not-regulated in LR when exposed to salinity stress. Regarding flavonol-3-O-glucoside L-rhamnosyltransferase (*Cluster-40,571.163208*) and kaempferol 3-O-beta-D-galactosyltransferase (*Cluster-40,571. 188,476*), their abundance of transcripts were higher in LC than LR, with expression up-regulated in LC yet non-regulated in LR under salinity stress. Besides, some other genes—including the novel plant SNARE (*Cluster-40,571.242780*), flavonoid 3′-monooxygenase (*Cluster-40,571.294284*), and flavonoid 3′-monooxygenase-like (*Cluster-40,571.294288*), showed consistently greater expression in LR than LC, with transcription levels up-regulated in LR but down-regulated or not regulated in LC when the plants were exposed to salinity stress (Fig. 11b). Further, for most of the changed metabolites in flavone and flavonol biosynthesis pathway under salinity stress in wolfberry, their content stayed at a higher level in LR but a lower level in LC. As shown in Fig. 11c, the 3,7-di-O-methylquercetin continued to have a lower content under the control condition, but this was up-regulated to greater extent in LC and down-regulated in LR when exposed to salinity stress. Moreover, the content of isovitexin, astragalin, and cosmosiin stayed at higher level in LR than LC under control condition, yet down-regulated both in LR and LC under salinity stress. The rutin content was found higher in LR than LC, and this did not change under salinity stress. The cynaroside content remained at a lower statement in LC but higher in LR under control condition, while it was down-regulated in LC and up-regulated in LR under high salinity condition (Fig. 11c).

## Discussion

Soil salinity will continue to threaten crop production and security in the future. Cultivation of salt-tolerant crops is therefore the most effective way to overcome this pressing environmental problem. In this study, we found that black wolfberry was more resistive to salinity than Chinese wolfberry.

### ABA content is normally low but increases sharply when exposed to high salinity in LR

ABA is involved in the growth and development regulation of plants, such as inhibiting seed germination, promoting dormancy, causing stomatal closure, inhibiting growth, and promoting leaf senescence and shedding [45]. At the same time, it also plays a vital role in coping with a variety of stresses that plants might incur in nature [46]. ABA is a kind of hormone which stay at a low content but with great effects in plants. Under stressful condition, the ABA concentration will increase and induce changes in certain metabolic processes in plants and play a role in resistance to adversity. Salinity stress leads to much ABA accumulating in plant roots, which is transported to the above ground parts via xylem fluid flow. As a result, ABA accumulation in leaves delays the leaf expansion rate and promotes stomatal closure, which reduces the transpiration rate of plants and the transport of salt in root caps, thus alleviating the damage to plants caused by salinity stress [47].

Comparing LC and LR, we found that the ABA content in LR was significantly lower than that in LC under non-stressed growing condition. When exposed to the high salinity condition, the LR quickly accumulated a large amount of ABA to resist salinity stress, but only a small amount of ABA was increased in LC to withstand injury from high salinity. These results confirm the strong resistance to salinity stress of black wolfberry.

### Flavone and flavonoid stay at a higher content under non-stressed condition than salinity stress in LR

Flavonoids constitute a class of important secondary metabolites widely found in plants, and they can affect many traits of plants [48]. Flavonoids play a key role in plant growth and protection against extreme environments [49, 50]. The type, content, distribution, and function of particular flavonoids vary greatly among species but also different among plant tissues and even across development stages. During the growth and development of a plant, the metabolites of flavonoids are in a process of dynamic change [51–53]. Many studies have reported that the metabolism of flavonoids is involved in plants responding to biotic and abiotic stress, such as salinity stress, oxidative stress, drought stress, insect chewing, and others [54–56]. Flavonoids can improve the adaptability of plants to adversity by eliminating the accumulation of ROS, or cooperate with other stress response factors (such as ABA and GA) to mitigate the damage caused by adverse biotic or abiotic factors [57–59].

In this paper, through the joint analysis of transcriptome and metabolome data, we find that irrespective of exposure to salinity stress or not, flavonoids and flavonoids were occurred at significantly higher content in LR than in LC, which likely promoted the higher salinity tolerance of LR than LC. In addition, via comparing their content in leaves under salinity stress and normal grow conditions, it was found that the accumulation of flavonoids and flavonoids in LC was significantly augmented under high salinity stress, indicating that LC need more flavones to resist the harm caused by high salinity. However, high salinity stress did not likewise induce a large accumulation of flavonoids in LR, perhaps it is because that the already higher content of flavonoids under normal condition, which was sufficient to resist high salinity stress. This would also explain the higher tolerance of black wolfberry to salinity stress.

## Conclusions

To sum up, through this study, we have shown that LR is ready to defence high salinity stress under normal condition, due to a lower ABA content and a higher level of flavonoids. Furthermore, when exposed to a high salinity environment, these LR plants will improve their resistance by accumulating much ABA on one hand, and on the other hand, mitigate oxidative damage which caused by high salinity via the high level of flavonoids already presented in their tissues. This research not only reveals the intrinsic reason why the black wolfberry tolerant to high salinity stress, but also suggests that to enhance the salinity tolerance of Chinese wolfberry by improving its flavonoids level, through in vivo or in vitro *techniques*, which would also simultaneously increase the nutritional value of its fruits and leaves.

## Methods

### Plant materials and growth conditions

Seedlings used in the experiments were cultured from twigs cuttage of Chinese wolfberry (*Lycium chinese*, LC*)* and black wolfberry (*L. ruthenicum*, LR*)*. Both Chinese wolfberry and black wolfberry were gained from the *Lycium barbarum* germplasm nursery in Ningxia, China. All the wolfberry seeds were surface-sterilized with 25% sodium hypochlorite, and then air-dried inside a horizontal laminar-flow hood, before sowing them on Murashige and Skoog(MS) medium which containing 1% sucrose and 0.8% agar. After stratification at 4 °C in dark for 3 days, the seed plates were transferred into a 10 h-light/14 h-dark photoperiod growth chamber whose temperature was controlled at 25 °C. Then, the seed germination rate of each plate was recorded every day from 1st day to 24th day. In addition, the wolfberry twigs were sterilized successively by 0.1% mercuric chloride, 70% ethyl alcohol, sterile water, and dried inside a horizontal laminar-flow hood subsequently. Then cut into the MS medium containing NaCl or not, which finally moved into the growth chamber to continue growing. After growing 21 days, the leaves were collected respectively, frozen into liquid nitrogen immediately, and stored in the − 80 °C refrigerator for later detection using.

### Phenotype characterization

The salinity tolerance of each wolfberry species (LC and LR) was determined experimentally. Firstly, to assess this phenotypic trait at germination stage, sterilized seeds of the two species were germinated on MS medium containing 150 mM of NaCl or control. The germination rate of *LC* and *LR* seeds were recorded after their stratification and development in the growth chamber for a few days. To evaluate salt tolerance at the vegetative growth stage, tissue-cultured seedlings of both wolfberry were cut and inserti into MS medium containing 150 mM NaCl or control, followed by their transferal to the growth chamber to grow continuously.

### Phytohormones ABA, JA and SA quantifications

For ABA, JA and SA content measurement, the wolfberry leaves were harvested, weighted, and immediately frozen into liquid nitrogen, and stored at − 80 °C until needed. Then the leaf tissues (50 mg fresh weight) were grounded to fine powder in liquid nitrogen, and extracted with extraction buffer containing methanol/water/formic acid (15:4:1,V/V/V). The combined extracts were evaporated to dryness under nitrogen gas stream, reconstituted in 80% methanol (V/V), and filtrated (PTFE, 0.22 μm; Anpel) before LC-MS/MS analysis. Nextly, the sample extracts were analyzed using an LC-ESI-MS/MS system (HPLC, Shim-pack UFLC SHIMADZUCBM30A system, www.shimadzu.com.cn/; MS, Applied Biosystems 6500 Triple Quadrupole, www.appliedbiosystems.com.cn/). The analytical conditions were as follows, HPLC: column, Waters ACQUITY UPLC HSS T3 C18 (1.8 μm, 2.1 mm*100 mm). The solvent system was composed of water (0.05% acetic acid) and acetonitrile (0.05% acetic acid), the gradient program was carried out as follows, which is water (0.05% acetic acid): acetonitrile (0.05% acetic acid) (95:5 V/V) at 0 min, water (0.05% acetic acid): acetonitrile (0.05% acetic acid) (95:5 V/V) at 1 min, water (0.05% acetic acid): acetonitrile (0.05% acetic acid) (5:95 V/V) at 8 min, water (0.05% acetic acid): acetonitrile (0.05% acetic acid) (5:95 V/V) at 9 min, water (0.05% acetic acid): acetonitrile (0.05% acetic acid) (95:5 V/V) at 9.1 min, water (0.05% acetic acid): acetonitrile (0.05% acetic acid) (95:5 V/V) at 12 min; the flow rate was 0.35 mL/min; the temperature was 40 °C; the injection volume was 2 μL. The effluent was alternatively connected to an ESI-triple quadrupole-linear ion trap (QTRAP)-MS.AB 6500 QTRAP LC/MS/MS System, equipped with an ESI Turbo Ion-Spray interface, operating in both positive and negative ion modes and controlled by Analyst 1.6 software (AB Sciex). The ESI source operation parameters were as follows: ion source, turbo spray; source temperature was 500 °C; ion spray voltage (IS) was 4500 V; curtain gas (CUR) were set at 35.0 psi; the collision gas (CAD) was medium. DP and CE for individual MRM transitions was done with further DP and CE optimization. A specific set of MRM transitions was monitored for each period according to the plant hormones eluted within this period. Three replicates of each assay were performed [60–63].

### RNA extractions

Total RNA was extracted from detached leaves with the Trizol reagent (Invitrogen) according to the manufacturer protocol. The extracted total RNA was treated with RNase-free DNase I (Thermo Scientific) to remove any trace amounts of DNA contamination. Next, the quality and quantity of extracted RNA were determined by measuring the absorbance at A260/A280 and A260/A230 in a spectrophotometer (NanoPhotometer). RNA integrity and its absence of DNA contamination were further verified by agarose gel electrophoresis. The concentration of RNA in a given sample was measured with high accuracy by a fluorimeter (Qubit 2.0), and the integrality of RNA was precisely confirmed by a bioanalyzer (Agilent 2100).

### cDNA library construction and sequencing

The mRNA was acquired in two ways: firstly, the mRNA with a polyA tail were enriched by the Oligo(dT) magnetic beads, and secondly, mRNA was obtained by removing rRNA from total RNA. Afterwards, RNA strands were broken into short fragments in a fragmentation buffer. These short-RNA fragments served as a template to synthesize the first strand cDNA with random hexamers. The second strand cDNA was synthesized by dNTPs (dUTP, dATP, dGTP, dCTP), DNA polymerase I, and first strand cDNA immersed together in a buffer solution. The ensuing double-stranded cDNA was purified by AMPure XP beads, after which the tail of purified double-strand cDNA was repaired, a polyA tail added, and the sequencing joint connected. Patterns were then picked by AMPure XP beads, and the final cDNA library acquired via PCR enrichment. Next, the final cDNA library was determined, mainly quantified by Qubit 2.0 and its insert size detected by an Agilent 2100; the effective concentration was measured accurately by qPCR. After the cDNA library was determined to be qualified, sequencing was carried out using Illumina Hi-Seq.

### Transcript splicing

Clean reads were gained after sequencing, filtration, error rate checking, and GC content-distribution checking. These clean reads were assembled to derive the reference sequence used later, by using Trinity software. The transcriptomes were hierarchically clustered by Corset (https://code.google.com/p/corset-project/), a software tool designed for obtaining gene-level counts from any de novo transcriptome assembly, from which the longest cluster sequence was designated the unigene for later analysis. This work was performed in the Metware company (http://www.metware.cn/).

### Gene annotations

Using BLAST software, each unigene was compared with several public databases: KEGG (Kyoto Encyclopedia of Genes and Genomes), NR (NCBI non-redundant protein sequences), Swiss-Prot (manually annotated and reviewed protein sequences), GO (Gene Ontology), and KOG/COG (Clusters of Orthologous Groups of proteins). The amino acid sequence of a given unigene was predicted, followed by blasting it against the Pfam (Protein family) database using HMMER software.

### Differentially expressed genes (DEGs)

To explore the profiles of DEGs between LR and LC under different salinity conditions, we analyzed gene expression patterns via DESeq2, to obtain robust DEGs sets. The identification of statistically significant DEGs and their respective fold-changes in gene expression level were implemented by an R package. After doing this, the multiple hypothesis testing was performed by first correcting the *P*-value of each gene to control the False Discovery Rate (FDR), using the Benjamini-Hochberg procedure. The criteria used for identifying DEGs were a |log_2_fold change| ≥ 1 with an FDR < 0.05.

### Functional annotation of DEGs

A cluster analysis of gene expression pattern was done to predict the genes functions and determine their distribution frequency across functional categories. This analysis relied on annotating genes to KEGG database to identify significantly enriched metabolic pathways or signal transduction pathways in DEGs versus the whole-genome background. Gene Ontology (GO) is another way to analyze gene sets, by describing the functioning of DEGs in terms of molecular function, biological progress and cellular component.

### Metabolomics

The detached leaves of wolfberry plants (LC and LR) were freeze-dried and ground in a mixer mill. A 100 mg powder subsample was extracted in 1.2 ml of 70% aqueous methanol at 4 °C overnight, followed by centrifuging at 10000 *g* for 10 min; the ensuing supernatant was absorbed and passed through 0.22-μm pore size filter before its UPLC-MS (Ultra Performance Liquid Chromatography - Tandem Mass Spectrometry) analysis. Then, each extract sample was analyzed in an UPLA-ESI-MS/MS system (UPLC, Shim-pack UFLC SHIMADZU CBM30A system, www.shimadzu.com.cn/;MS, Applied Biosystems. 6500 Q TRAP, www.appliedbiosystems.com.cn/). This work was performed in the Metware company.

### Screening of differential metabolites

Differential metabolites should be excavated accurately from multiple perspectives, by applying both univariate and multivariate statistical analyses. The differential metabolites in the wolfberry species (LC and LR) under salinity conditions could be screened out, in a preliminary way, by the variable importance in projection (VIP) value based on the OPLS-DA (Orthogonal Partial Least Squares - Discriminant Analysis) results. The threshold values used for screening would assign a significant difference when the fold change ≥2 or ≤ 0.5, in addition to having a VIP ≥ 1.

### Enrichment analysis and functional annotation of differential metabolites

The differential metabolites were annotated using the KEGG database and enriched by KEGG pathway, according to the results from the preceding differential metabolites analysis. The rich factor is a ratio of the number of differential metabolites in corresponding pathway to the total number of metabolites detected and annotated in that pathway. Accordingly, the enrichment degree is inferred to be higher when this ratio has a larger value, In addition, the closer its *P*-value was to zero, the more outstanding was a given enrichment.

### Statistical analysis

All experiments were carried out at least three times, independently, with similar results. All values are presented as means ± SD. Statistical significance was based on unpaired two-sample Student’s *t*-tests, as determined in Sigmaplot 10 software. Principal component analysis (PCA) was also performed on all data sets.

## Supplementary Information

**Additional file 1: Figure S1**. The press marker data in LC and LR under salinity stress. a The Na^+^ content. b The K^+^ content. c The K^+^/Na^+^ ratio. d Fv/Fm. e The MDA content. f The GSH content. g The proline content. h The amino acid content.

## Data Availability

The datasets generated and analysed during the current study are available in the supplementary information files. The link address of transcriptome data is https://dataview.ncbi.nlm.nih.gov/object/PRJNA666311?reviewer=ko1g8cgo4piqpi2d4fje9oaf79.

## Abbreviations

LC: *Lycium. Chinese*
LR: *Lycium. Ruthenicum*
ABA: Abscisic acid
H_2_O_2_: hydrogen peroxide
JA: Jasmonic acid
ROS: Reactive oxygen species
SA: Salicylic acid
MS: Murashige and Skoog
VIP: Variable importance in prejection
MDA: Malondialdehyde
TBA: Thiobarbituric acid
TCA: Trichloroacetic acid
DEGs: Differentially expressed genes
PCA: Principal component analysis
KEGG: Kyoto Encyclopedia of Genes and Genomes

## References

[1] FAO. The state of the world’s land and water resources for food and agriculture (SOLAW)—managing systems at risk. Food and Agriculture Organization of the United Nations and Earthscan. London: Rome and Earthscan; 2011. http://www.fao.org/ag/agl/agll/spush.

[2] Wegner LH, Stefano G, Shabala L, Rossi M, Mancuso S, Shabala S (2011). Sequential depolarization of root cortical and stelar cells induced by an acute salt shock implications for Na^+^ and K^+^ transport into xylem vessels. Plant Cell Environ.

[3] Cheeseman JM (2013). The integration of activity in saline environments: problems and perspectives. Funct Plant Biol.

[4] Benito B, Haro R, Amtmann A, Cuin TA, Dreyer I (2014). The twins K^+^ and Na^+^ in plants. J Plant Physiol.

[5] Wu HH, Zhang XC, Giraldo JP, Shabala S (2018). It is not all about sodium: revealing tissue specificity and signalling roles of potassium in plant responses to salt stress. Plant Soil.

[6] Bazihizina N, Colmer TD, Cuin TA, Mancuso S, Shabala S (2019). Friend or foe? Chloride patterning in halophytes. Trends Plant Sci.

[7] Miller G, Suzuki N, Ciftci-Yilmaz S, Mittler R (2010). Reactive 1645 oxygen species homeostasis and signalling during drought and salinity stresses. Plant Cell Environ.

[8] Ma L, Zhang H, Sun L, Jiao Y, Zhang G, Miao C, Hao F (2012). NADPH oxidase AtrbohD and AtrbohF function in ROS-dependent regulation of Na^+^/1614 K^+^ homeostasis in Arabidopsis under salt stress. J Exp Bot.

[9] Noctor G, Foyer CH (1998). Ascorbate and glutathione: keeping active oxygen under control. Annu Rev Plant Physiol Plant Mol Biol.

[10] Barhoumi Z, Djebali W, Chaïbi W, Abdelly C, Smaoui A (2007). Salt impact on photosynthesis and leaf ultrastructure of Aeluropus littoralis. J Plant Res.

[11] Pottosin I, Shabala S (2016). Transport across chloroplast membranes: optimizing photosynthesis for adverse environmental conditions. Mol Plant.

[12] Dodd AN, Kudla J, Sanders D (2010). The language of calcium signaling. Annu Rev Plant Biol.

[13] Knight H, Trewavas AJ, Knight MR (1997). Calcium signalling in Arabidopsis thaliana responding to drought and salinity. Plant J.

[14] Jiang Z, Zhou X, Tao M, Yuan F, Liu L, Wu F, Wu X, Xiang Y, Niu Y, Liu F (2019). Plant cell-surface GIPC sphingolipids sense salt to trigger Ca^2+^ influx. Nature.

[15] Laohavisit A, Richards SL, Shabala L, Chen C, Colaço RDDR, Swarbreck SM, Shaw E, Dark A, Shabala S, Shang Z (2013). Salinity-induced calcium signaling and root adaptation in Arabidopsis require the calcium regulatory protein annexin1. Plant Physiol.

[16] Stephan AB, Kunz HH, Yang E, Schroeder JI (2016). Rapid hyperosmotic-induced Ca^2+^ responses in Arabidopsis thaliana exhibit sensory potentiation and involvement of plastidial KEA transporters. Proc Natl Acad Sci U S A.

[17] Feng W, Kita D, Peaucelle A, Cartwright HN, Doan V, Duan Q, Liu M-C, Maman J, Steinhorst L, Schmitz-Thom I (2018). The FERONIA receptor kinase maintains cell wall integrity during salt stress through Ca^2+^ signaling. Curr Biol.

[18] Zhao C, Zayed O, Yu Z, Jiang W, Zhu P, Hsu C-C, Zhang L, Tao WA, Lozano-Durán R, Zhu J-K (2018). Leucine-rich repeat extensin proteins regulate plant salt tolerance in Arabidopsis. Proc Natl Acad Sci U S A.

[19] Boudsocq M, Barbier-Brygoo H, Laurière C (2004). Identification of nine sucrose nonfermenting 1-related protein kinases 2 activated by hyperosmotic and saline stresses in Arabidopsis thaliana. J Biol Chem.

[20] Fujii H, Verslues PE, Zhu J-K (2011). Arabidopsis decuple mutant reveals the importance of SnRK2 kinases in osmotic stress responses in vivo. Proc Natl Acad Sci U S A.

[21] McLoughlin F, Galvan-Ampudia CS, Julkowska MM, Caarls L, Van Der Does D, Laurière C, Munnik T, Haring MA, Testerink C (2012). The Snf1-related protein kinases SnRK2.4 and SnRK2.10 are involved in maintenance of root system architecture during salt stress. Plant J.

[22] Chan KX, Phua SY, Crisp P, McQuinn R, Pogson BJ (2016). Learning the languages of the chloroplast: retrograde signaling and beyond. Annu Rev Plant Biol.

[23] Chaves MM, Flexas J, Pinheiro C (2009). Photosynthesis under drought and salt stress: regulation mechanisms from whole plant to cell. Ann Bot.

[24] Bose J, Munns R, Shabala S, Gilliham M, Pogson B, Tyerman SD (2017). Chloroplast function and ion regulation in plants growing on saline soils: lessons from halophytes. J Exp Bot.

[25] Xiong L, Lee H, Ishitani M, Zhu JK (2002). Regulation of osmotic stress-responsive gene expression by the LOS6/ABA1 locus in Arabidopsis. J Biol Chem.

[26] Jia W (2002). Salt-stress-induced ABA accumulation is more sensitively triggered in roots than in shoots. J Exp Bot.

[27] Fricke W, Akhiyarova G, Veselov D, Kudoyarova G (2004). Rapid and tissue-specific changes in ABA and in growth rate in response to salinity in barley leaves. J Exp Bot.

[28] Zhang FP, Sussmilch F, Nichols DS, Cardoso AA, Brodribb TJ, McAdam SAM (2018). Leaves, not roots or floral tissue, are the main site of rapid, external pressure-induced ABA biosynthesis in angiosperms. J Exp Bot.

[29] Nath M, Bhatt D, Jain A, Saxena SC, Saifi SK, Yadav S, Negi M, Prasad R, Tuteja N (2019). Salt stress triggers augmented levels of Na^+^, Ca^2+^ and ROS and alter stress responsive gene expression in roots of CBL9 and CIPK23 knockout mutants of Arabidopsis thaliana. Environ Exp Bot.

[30] Mittler R, Blumwald E (2015). The roles of ROS and ABA in systemic acquired acclimation. Plant Cell.

[31] Zhang Y, Tan J, Guo Z, Lu S, He S, Shu W, Zhou B (2009). Increased abscisic acid levels in transgenic tobacco over-expressing 9 cis-epoxycarotenoid dioxygenase influence H_2_O_2_ and NO production and antioxidant defences. Plant Cell Environ.

[32] Apse MP, Blumwald E (2002). Engineering salt tolerance in plants. Curr Opin Biotechnol.

[33] Munns R (2002). Comparative physiology of salt and water stress. Plant Cell Environ.

[34] Verslues PE, Agarwal M, Katiyar-Agarwal S, Zhu J, Zhu JK (2006). Methods and concepts in quantifying resistance to drought, salt and freezing, abiotic stresses that affect plant water status. Plant J.

[35] Henry C, Bledsoe SW, Griffiths CA, Kollman A, Paul MJ, Sakr S, Lagrimini LM (2015). Differential role for trehalose metabolism in salt-stressed maize. Plant Physiol.

[36] Mansour MMF, Ali EF (2017). Evaluation of proline functions in saline conditions. Phytochemistry.

[37] Ashraf M, Foolad MR (2007). Roles of glycine betaine and proline in improving plant abiotic stress resistance. Environ Exp Bot.

[38] Verbruggen N, Hermans C (2008). Proline accumulation in plants: a review. Amino Acids.

[39] Ben Rejeb K, Abdelly C, Savouré A (2014). How reactive oxygen species and proline face stress together. Plant Physiol Biochem.

[40] Jayakannan M, Bose J, Babourina O, Rengel Z, Shabala S (2013). Salicylic acid improves salinity tolerance in Arabidopsis by restoring membrane potential and preventing salt induced K^+^ loss via a GORK channel. J Exp Bot.

[41] Zhang Z, He K, Zhang T, Tang D, Li R, Jia S (2019). Physiological responses of goji berry (*Lycium barbarum* L.) to saline-alkaline soil from Qinghai region, China. Sci Rep.

[42] Rao S, Tian Y, Xia X, Li Y, Chen J (2020). Chromosome doubling mediates superior drought tolerance in *Lycium ruthenicum* via abscisic acid signaling. Horticulture Res.

[43] Wu D, Ji J, Wang G, Guan C, Jin C (2014). LchERF, a novel ethylene-responsive transcription factor from Lycium chinense, confers salt tolerance in transgenic tobacco. Plant Cell Rep.

[44] Song X, Diao J, Ji J, Wang G, Guan C, Jin C, Wang Y (2016). Molecular cloning and identification of a flavanone 3-hydroxylase gene from Lycium chinense, and its overexpression enhances drought stress in tobacco. Plant Physiol Biochem.

[45] Wasilewska A, Vlad F, Sirichandra C (2008). An update on abscisic acid signaling in plants and more [J]. Mol Plant.

[46] Dar NA, Amin I, Wani W (2017). Abscisic acid: Akey regulator of abiotic stress tolerance in plants [J]. Plant Gene.

[47] Hartung W, Schraut D, Jiang F (2005). Physiology of abscisic acid (ABA) in roots under stress--a review of the relationship between root ABA and radial water and ABA flows [J]. Aust J Agric Res.

[48] Seymour GB, Chapman NH, Chew BL, Rose JK (2013). Regulation of ripening and opportunities for control in tomato and other fruits. Plant Biotechnol J.

[49] Dicko MH, Gruppen H, Barro C (2005). Impact of phenolic compounds and related enzymes in Sorghum varieties for resistance and susceptibility to biotic and abiotic stresses. J Chem Ecol.

[50] Petrussa E, Braidot E, Zancani M, Peresson C, Bertolini A, Patui S, Vianello A (2013). Flavonoids-biosynthesis, transport and involvement in stress responses. Int J Mol Sci.

[51] Zu Y, Chen H, Wang W, Jia J, Zhu L, Zhang N (2006). Sugar, phytohormone, tannins and flavonoids changes during the growth and development of *Eupatorium adenophorum*. Bull Bot Res.

[52] Makoi J, Ndakidemi P (2011). Changes in plant growth, nutrient dynamics and accumulation of flavonoids and anthocyanins by manipulating the cropping systems involving legumes and cereals. Aust J Agric Eng.

[53] Choi SH, Ahn JB, Kim HJ, Im NK, Kozukue N, Levin CE, Friedman M (2012). Changes in free amino acid, protein, and flavonoid content in jujube (Ziziphus jujube) fruit during eight stages of growth and antioxidative and cancer cell inhibitory effects by extracts. J Agric Food Chem.

[54] Mahajan M, Yadav SK (2014). Overexpression of a tea flavanone 3-hydroxylase gene confers tolerance to salt stress and alternaria solani, in transgenic tobacco. Plant Mol Biol.

[55] Luo P, Shen Y, Jin S, Huang S, Cheng X, Wang Z, Li P, Zhao J, Bao M, Ning G (2016). Overexpression of *Rosa rugosa* anthocyanidin reductase enhances tobacco tolerance to abiotic stress through increased ROS scavenging and modulation of ABA signaling. Plant Sci.

[56] Zhu LJ, Deng XG, Zou LJ, Zhang DW, Lin HH (2017). Enhancement of stress tolerance in cucumber seedlings by proanthocyanidins. Biol Plant.

[57] Steyn WJ, Wand SJE, Holcroft DM, Jacobs G (2002). Anthocyanins in vegetative tissues: a proposed unified function in photoprotection. New Phytol.

[58] Li S, Wang W, Gao J, Yin K, Wang R, Wang C, Petersen M, Mundy J, Qiu J (2016). MYB75 phosphorylation by MPK4 is required for light-induced anthocyanin accumulation in Arabidopsis. Plant Cell.

[59] Zhao C, Zhang H, Song C, Zhu J-K, Shabala S. Mechanisms of plant responses and adaptation to soil salinity. The Innovation. 2020. 10.1016/j.xinn.2020.100017. 10.1016/j.xinn.2020.100017 PMC8454569 34557705

[60] Šimura J, Antoniadi I, Široká J (2018). Plant Hormonomics: multiple Phytohormone profiling by targeted metabolomics. Plant Physiol.

[61] Floková K, Tarkowská D, Miersch O (2014). UHPLC-MS/MS based target profiling of stressinduced phytohormones. Phytochemistry.

[62] Kun C, Ya L, Xi Z, et al. Comparison of sample pretreatment methods for the determination of multiple phytohormones in plant samples by liquid chromatography-electrospray ionization-tandem mass spectrometry. Microchem J. 2015;121:25–31.

[63] Hua X, Wen C, Tian Y, et al. Spatio-temporal profiling of abscisic acid, indoleacetic acid and jasmonic acid in single rice seed during seed germination. Anal Chim Acta. 2018;1031:119–27. 10.1016/j.aca.2018.05.055 30119729

