# Correction to: Metabolomic and transcriptomic analysis of *Lycium chinese* and *L. ruthenicum* under salinity stress

**DOI:** 10.1186/s12870-022-03436-9

**Published:** 2022-01-24

**Authors:** Xiaoya Qin, Yue Yin, Jianhua Zhao, Wei An, Yunfang Fan, Xiaojie Liang, Youlong Cao

**Affiliations:** Wolfberry Science Institute, Ningxia Academy of Agriculture and Forestry Sciences / National Wolfberry Engineering Research Center, Yinchuan, 750002 China

**Correction to: BMC Plant Biol 22, 8 (2022)**

**https://doi.org/10.1186/s12870-021-03375-x**

Following publication of the original article [1], it was noted that due to a typesetting error Supplementary Fig. S1 was mistakenly processed as Fig. 1.

**Fig. 1:**
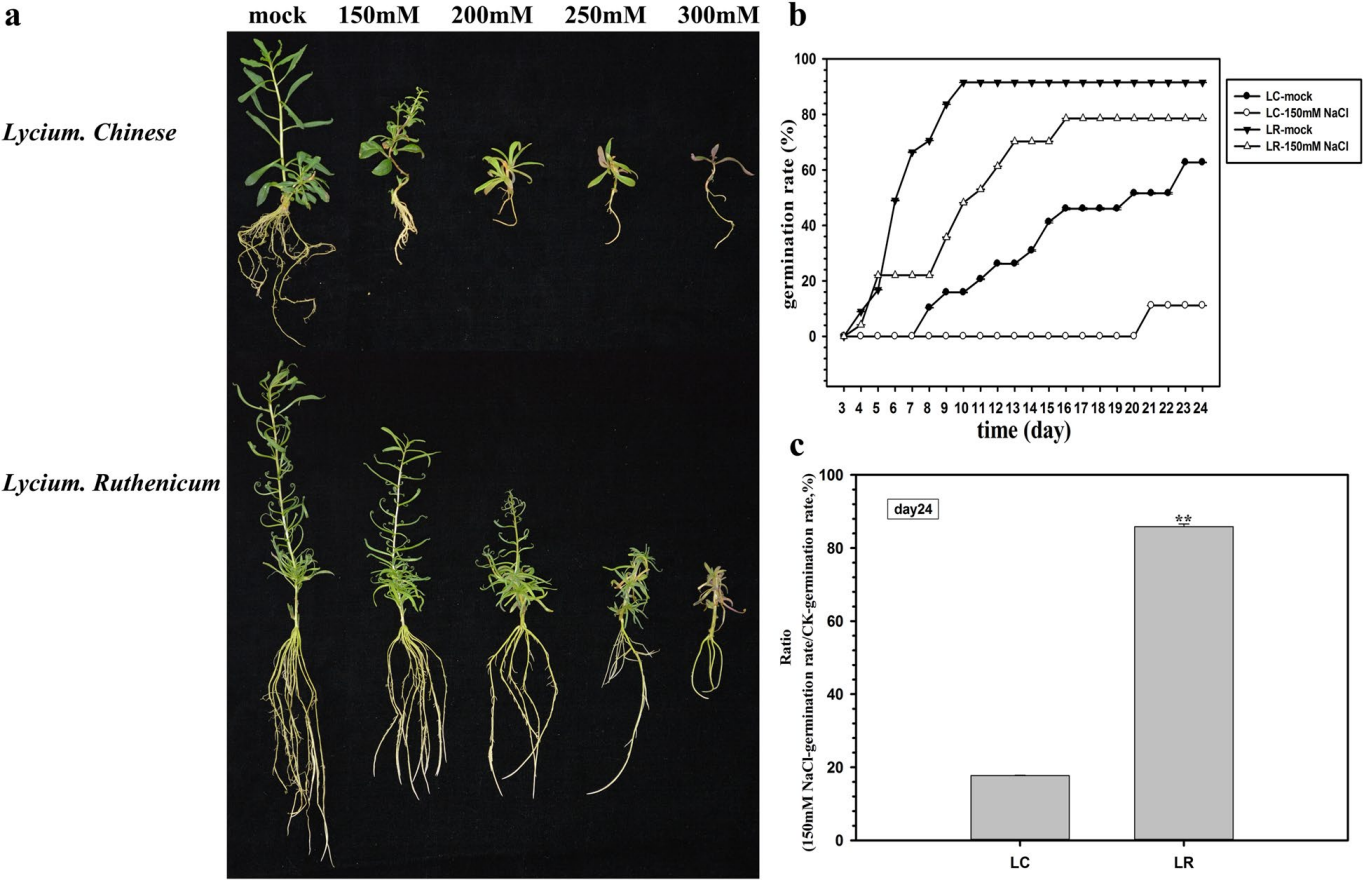
Phenotype analysis of *Lycium chinese* and *L. ruthenicum* under salinity stress. **a** The twig cuttings of *L. chinese* and *L. ruthenicum* were planted in the MS medium containing 0 mM (CK), 150 mM, 200 mM, 250 mM or 300 mM of NaCl. Pictures were taken after 3 weeks of cultivation. **b** Seed germination rate of *L. chinese* and *L. ruthenicum* under control and 150 mM NaCl concentrations was calculated from day 3 to day 24. **c** The ratio of germination rate 150 mM NaCl/ CK was calculated at day 24 after seeds were sown. * *P* < 0.05, ** *P* < 0.01. *N* = 3. Bars = means ± SEM

The correct figure is included in this correction, and the original article [1] has been corrected.
