# Supplementary figures and images for: Metabolomic and transcriptomic analysis of Lycium chinese and L. ruthenicum under salinity stress
Source: BMC Plant Biol. 2022 Jan 3;22:8. doi: 10.1186/s12870-021-03375-x (PMC8722043; doi:10.1186/s12870-021-03375-x)

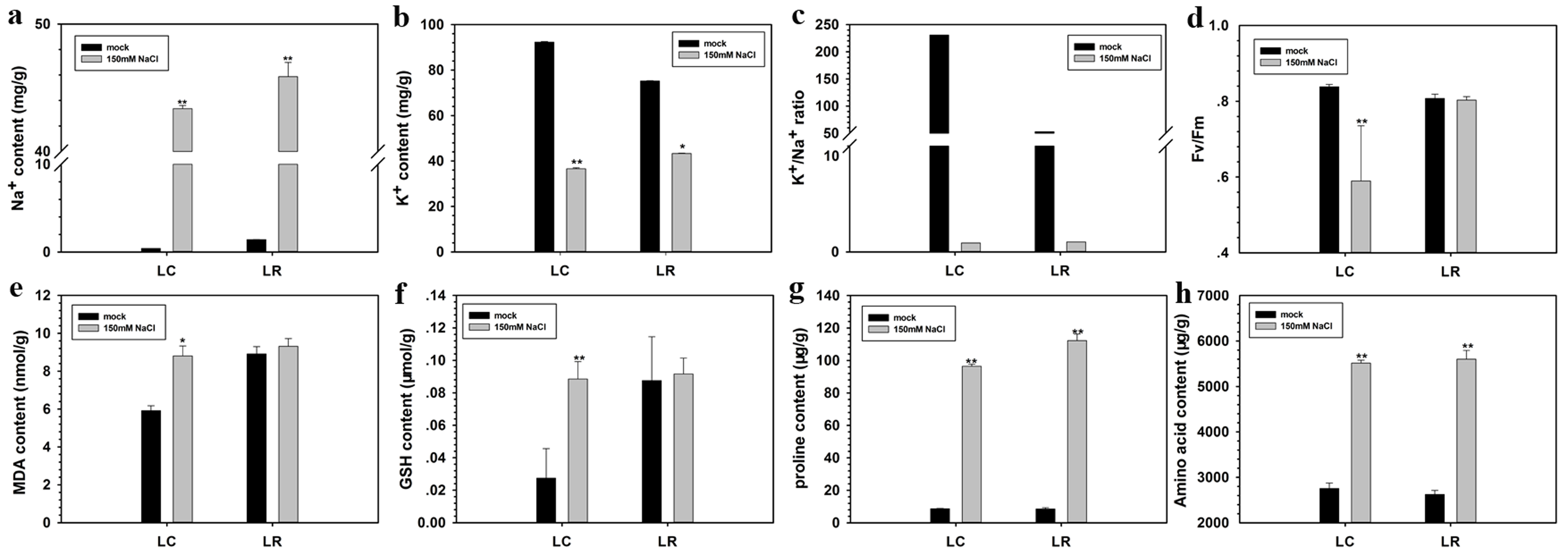

Supplement: Supplementary file 1 — Additional file 1: Figure S1. The press marker data in LC and LR under salinity stress. a The Na+ content. b The K+ content. c The K+/Na+ ratio. d Fv/Fm. e The MDA content. f The GSH content. g The proline content. h The amino acid content. [file 12870_2021_3375_MOESM1_ESM.jpg]
